# The lipid-metabolic enzyme HSD17B12 drives lysosomal degradation of PD-L1 potentiating anti-tumor immunity in a mouse model

**DOI:** 10.1371/journal.pbio.3003603

**Published:** 2026-01-27

**Authors:** Zhihui Zhou, Ying Lu, Pan Li, Xin Liu, Wei Cheng, Hai-Ning Chen, Lunzhi Dai, Haiyan Ren

**Affiliations:** 1 Department of Pulmonary and Critical Care Medicine, Respiratory Infection and Intervention Laboratory of Frontiers Science Center for Disease-related Molecular Network, and State Key Laboratory of Biotherapy, West China Hospital of Sichuan University, Chengdu, Sichuan, China; 2 National Clinical Research Center for Geriatrics, State Key Laboratory of Biotherapy, West China Hospital, Sichuan University, Chengdu, Sichuan, China; 3 Colorectal Cancer Center, Department of General Surgery, West China Hospital, Sichuan University, Chengdu, Sichuan, China; Consejo Nacional de Investigaciones Científicas y Técnicas: Consejo Nacional de Investigaciones Cientificas y Tecnicas, ARGENTINA

## Abstract

The high prevalence of cancer immunotherapy resistance, coupled with substantial tumor heterogeneity, underscores the urgent need for innovative therapeutic targets. A deeper understanding of immunoregulatory mechanisms would provide new targets and combination therapeutic strategies for tumor therapy. In this study, we demonstrate that HSD17B12 enhances anti-tumor immunity and represents a promising therapeutic target. Mechanistically, HSD17B12 promotes lysosome-dependent degradation of PD-L1 via the VAC14 and ESCRT complexes across various malignancies, regardless of its 3-ketoacyl-CoA reductase activity. HSD17B12-deficient cells displayed PD-L1 accumulation in both tumor cells and exosomes, reducing T cell-mediated cytotoxicity. Notably, we found a significant negative correlation between HSD17B12 and PD-L1 expression in colorectal cancer tissues. Furthermore, high HSD17B12 expression in CRC correlated with increased infiltration of cytotoxic T cells. Based on these findings, we designed a peptide, HSD-CC1-NPGY, which effectively reduces PD-L1 expression in cells and suppresses tumor growth in a mouse model. Overall, our results establish HSD17B12 as an important regulator of anti-tumor immunity and a promising therapeutic target for cancer treatment.

## Abstract 2

Cancer immunotherapy faces widespread resistance driven by tumor heterogeneity and immune evasion through mechanisms like PD-L1 suppression. This study identifies the metabolic enzyme HSD17B12 as a regulator of PD-L1 degradation and shows that an HSD17B12-derived peptide boosts anti-tumor immunity in a mouse model.

## Introduction

Immune checkpoints play critical roles in regulating immune responses, particularly in cancer immunity. Targeting the immune checkpoints has shown significant clinical benefits across multiple cancers [1–6], such as melanoma, non-small-cell lung carcinoma (NSCLC), renal cell carcinoma (RCC), breast cancer, and colorectal cancer (CRC). Despite these advances, the overall response rate remains limited at 20%–40%, with many patients experiencing resistance or only short-term benefits [7–9].

Elucidating the regulatory mechanisms of immune checkpoints holds significant potential for advancing immune therapies [10–26]. AMPK has been shown to mediate the phosphorylation of PD-L1, triggering its ER-associated degradation (ERAD). Consequently, metformin has been explored for its potential in tumor immunotherapy by activating AMPK [24]. Additionally, PD-L1 palmitoylation by ZDHHC3/9 prevents its lysosomal targeting and degradation [14,27]. Inhibiting this palmitoylation—using 2-bromopalmitate (2-BP) or a designed peptide, CCP-S1—promotes PD-L1 degradation and enhances T cell-mediated cytotoxicity [27]. Moreover, Huntington-interacting protein 1-related (HIP1R) facilitates the lysosomal trafficking and degradation of PD-L1, thereby promoting T cell-mediated tumor killing [28]. Based on this mechanism, a fusion peptide named PD-LYSO—comprising the PD-L1-binding domain (Q776–Q807) and the lysosomal sorting signal (M966–Q979) of HIP1R—has been designed to efficiently reduce PD-L1 expression in tumor cells [28].

Despite progress in understanding immune checkpoint regulation, the heterogeneity of tumors leaves many regulatory mechanisms yet to be explored. Our study found that the very-long-chain 3-oxoacyl-CoA reductase, HSD17B12, regulates tumor immunity regardless of its reductase activity. HSD17B12 has been reported to play an important role in biological processes like steroid and lipid metabolism. It is highly expressed in several cancers, including ovarian, prostate, and breast cancers, and its pro-cancer mechanism might involve regulating lipid metabolism and hormone levels. In ovarian cancer, HSD17B12 silencing significantly inhibited tumor cell proliferation, while exogenous arachidonic acid restored the growth inhibitory effect, suggesting its function depends on the reductase activity [29]. Notably, HSD17B12 displayed a similar pro-carcinogenic phenotype in breast cancer and head and neck malignancies. Although HSD17B12 has long been known for its function in fatty acid elongation, its function and mechanism in tumor immunity have not been explored.

Here, we found that elevated HSD17B12 levels in CRC tissues are associated with increased cytotoxic T cell infiltration. Mechanistically, HSD17B12 boosts anti-tumor immunity by facilitating VAC14-ESCRT-mediated degradation of PD-L1, independently of CMTM6, HIP1R, and palmitoylation. Furthermore, we provided an effective strategy to degrade PD-L1 and suppress tumor growth in a mouse model. Collectively, our findings highlight HSD17B12’s role in tumor immunity and position it as a promising target for cancer therapy.

## Results

### HSD17B12 enhances cytotoxic T cell infiltration in colorectal cancer

**Fig 1:**
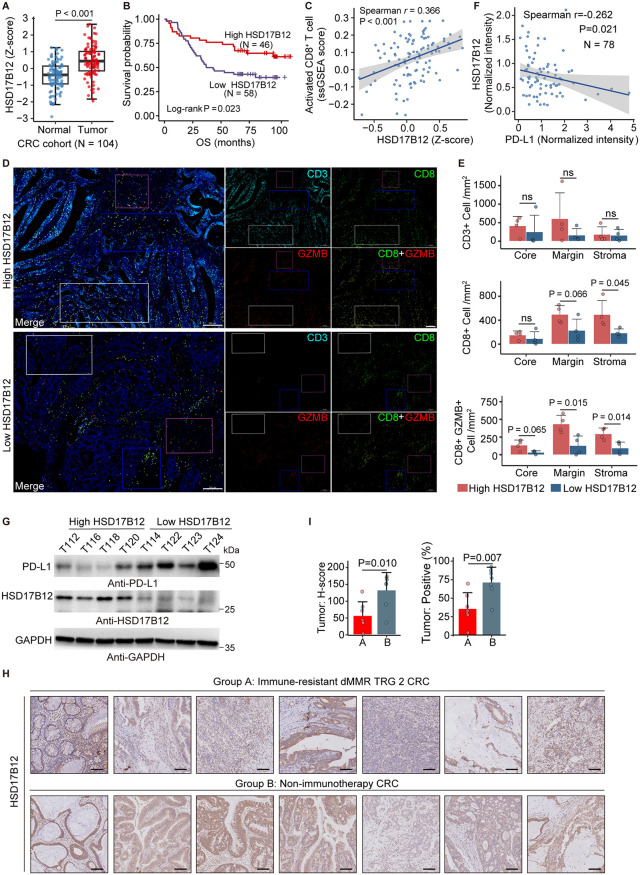
Clinical significance of HSD17B12 in colorectal cancer. **(A)** Box plot showing HSD17B12 protein expression in paired tumor tissues and distant normal tissues from CRC patients (Paired Wilcoxon signed-rank test). **(B)** Kaplan–Meier survival curve depicting the association between HSD17B12 protein expression in tumors and overall survival (log-rank test). **(C)** Scatter plot with a fitted line illustrating the relationship between HSD17B12 protein expression and the ssGSEA score for activated CD8^+^ T cells in primary tumors (Spearman correlation analysis). **(D)** Representative immunofluorescence images showing CD3, CD8, and GZMB staining in tumor samples. CD3^+^ indicates total T cells, CD8^+^ indicates CD8^+^ T cells, and CD8^+^GZMB^+^ indicates activated CD8^+^ T cells (scale bar: 200 µm). **(E)** Bar chart comparing immunofluorescence results between the high HSD17B12 and low HSD17B12 groups (Student *t* test). **(F)** HSD17B12 level correlates with PD-L1 expression in primary CRC tumors. Scatter plot with a fitted line showing the correlation between HSD17B12 and PD-L1 levels in primary tumors (Spearman correlation analysis). **(G)** High activated CD8^+^ T cell infiltration correlates low PD-L1 expression. Western blotting analysis of HSD17B12 and PD-L1 in tumor samples that were used for immunofluorescence analysis (D). **(H)** Immunohistochemistry images showing HSD17B12 expression in tumor tissues from 7 dMMR patients with TRG 2 (AJCC) pathology post-immunotherapy, compared to 7 patients who did not receive immunotherapy (scale bar: 100 µm). dMMR: deficient mismatch repair. **(I)** H-score and positive expression rate of HSD17B12 in tumor cells (Student *t* test). Numerical data of (A), (B), (C), (E), (F), and (I) can be found in S1 Data, sheet “Fig 1”.

Our analysis revealed that HSD17B12 expression was significantly elevated in CRC tumor tissue (Fig 1A) (S1 Table), and this upregulation was associated with a favorable prognosis (Fig 1B) (S2 Table). Single-sample gene set enrichment analysis (ssGSEA) of immune cells revealed a notably positive correlation between HSD17B12 expression and CD8^+^ T cell activation (Fig 1C), indicating that HSD17B12 may promote CD8^+^ T cell infiltration into tumors. CD8^+^ T cells are crucial mediators of anti-tumor immunity, eliminating cancer cells by secreting granzyme B and triggering apoptosis. The observed correlation underscores HSD17B12’s potential role in enhancing anti-tumor immune responses. Immunofluorescence analysis further supported this finding, showing increased infiltration of both CD8^+^ and activated granzyme B-positive (GzmB^+^) CD8^+^ T cells in tumors with elevated HSD17B12 expression (Fig 1D,1E). These results highlight HSD17B12 as a key regulator of T cell-mediated anti-tumor activity.

Furthermore, we analyzed 78 CRC tumor samples and found a strong negative correlation between HSD17B12 and PD-L1 expression (Fig 1F). Notably, the samples with higher levels of activated CD8^+^ T cell infiltration (Fig 1D) exhibited lower PD-L1 expression (Fig 1G). This implied that HSD17B12 plays a crucial role in reducing immune suppression by influencing PD-L1 expression.

Finally, we investigated the association between HSD17B12 expression levels and response to immunotherapy. Among dMMR CRC patients who were pathologically classified as tumor regression grade 2 (TRG2) after immunotherapy, HSD17B12 expression was significantly lower compared to the broader nonimmunotherapy CRC cohort (Fig 1H,1I). This finding suggests that HSD17B12 may influence immune response to immunotherapy in dMMR CRC tumors.

### HSD17B12 interacts with PD-L1 in living cells

**Fig 2:**
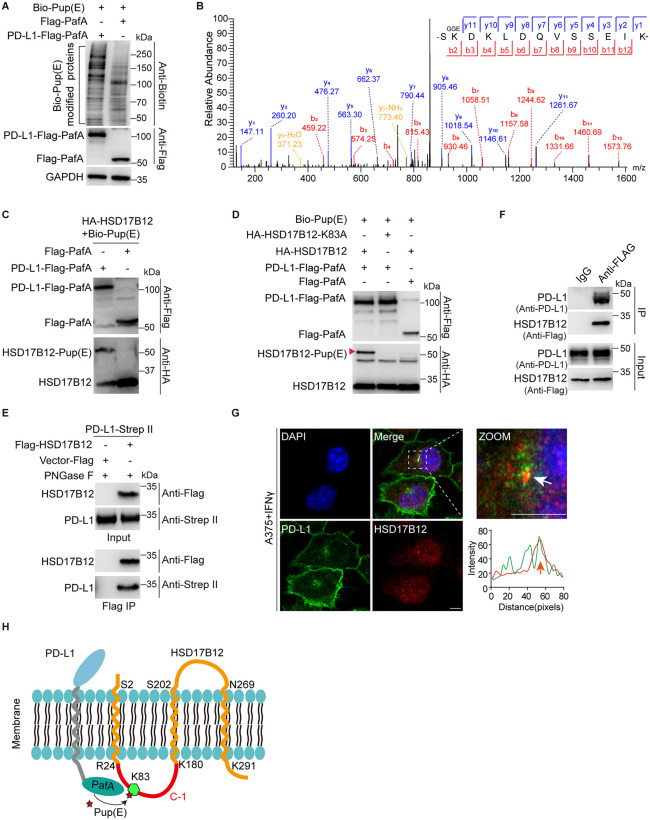
HSD17B12 interacts with PD-L1 in living cells. **(A)** Immunoblotting results of PD-L1’s interactome captured by the PUP-IT system. HEK293T cells were co-transfected with the PD-L1-Flag-PafA or Flag-PafA and Bio-Pup(E) plasmids. **(B)** Mass spectrum results showing K83 of HSD17B12 is labeled by PD-L1-Flag-PafA. **(C–E)** HSD17B12 interacts with PD-L1. (C) Cell lysates were separated on SDS-PAGE gels and detected using anti-Flag and anti-HA antibodies. Three biological replicates. (D) Immunoblotting showing PD-L1-Flag-PafA modifies the K83 residue of HSD17B12. Representative of three experiments. (E) HSD17B12 co-immunoprecipitates with PD-L1. Immunoblots for input and IP are shown. Three biological replicates. **(F)** Endogenous HSD17B12 co-immunoprecipitates with endogenous PD-L1. Immunoblots for input and IP are shown. **(G)** PD-L1 partially colocalizes with HSD17B12 in A375 cells. Representative images are shown (scale bars: 5 μm). Immunofluorescence staining was performed using the anti-PD-L1 and anti-HSD17B12 antibodies. **(H)** Schematic model showing the membrane localization of HSD17B12. Numerical data of (G) can be found in S1 Data, sheet “Fig 2”.

To identify potential PD-L1 regulators, we employed a proximity-tagging system called pupylation-based interaction tagging (PUP-IT) [30]. In this system, the bacterial Pup ligase, PafA, catalyzes the conjugation of Pup(E) to nearby lysine (Lys) residues on the bait and its associated partners (prey) (S1A Fig). To enable biotinylation within cells, Pup(E) was coupled with a bacteria-derived carboxylase domain. We then fused PafA to the C-terminus of PD-L1 (PD-L1-Flag-PafA), which localizes to the plasma membrane when glycosylated and primarily to the cytoplasm when not glycosylated (S1B Fig). Immunoblotting with anti-biotin antibodies showed a significant difference between the interactome of PD-L1-Flag-PafA and Flag-PafA (Fig 2A).

We performed denatured streptavidin-based purification from HEK293T cells co-expressing PD-L1-Flag-PafA and Bio-Pup(E), followed by LC-MS/MS analysis (S1A Fig). Proteins specifically labeled with an extra 243-Da mass (GGE modifications) on Lys residues in PD-L1-Flag-PafA samples but not in Flag-PafA samples were identified as potential PD-L1 binding partners (S1C Fig). Consistent with previous findings, we confirmed the modification of PGRMC1, a known PD-L1-interacting protein [31]. In addition, the very-long-chain 3-oxoacyl-CoA reductase (HSD17B12) was specifically modified in PD-L1-Flag-PafA samples, with the GGE modification localized to Lys83 on HSD17B12 (Fig 2B). To validate these findings, we co-expressed PD-L1-Flag-PafA, Pup(E), and HA-HSD17B12. Immunoblotting with anti-HA antibodies detected additional bands indicative of Pup(E) modification on HSD17B12 (Fig 2C). Substitution of HSD17B12 Lys83 with alanine (Ala) abolished this modification, confirming the modification site (Fig 2D). Furthermore, co-immunoprecipitation confirmed the interaction between HSD17B12 and PD-L1 in HEK293T cells, RKO colon cancer cells, and MDA-MB-231 triple-negative breast cancer cells (Figs 2E, S1D, S1E). To verify that endogenous HSD17B12 interacts with endogenous PD-L1, we employed CRISPR/Cas9 to knock-in a Flag tag at the C-terminus of the *HSD17B12* gene (S1F Fig). Subsequent co-immunoprecipitation confirmed that PD-L1 interacts with HSD17B12 under physiological conditions (Fig 2F). Immunofluorescence also revealed partial colocalization of PD-L1 and HSD17B12 in A375 melanoma cells and MDA-MB-231 cells (Figs 2G, S1G). Collectively, these findings demonstrate that HSD17B12 is an interaction partner of PD-L1.

Using the TMHMM-2.0 (https://services.healthtech.dtu.dk/service.php?TMHMM-2.0), we predicted that HSD17B12 contains three putative transmembrane domains (S1H Fig). Lys83 of HSD17B12, the PUP-IT modification residue, is positioned in the cytoplasmic area between two transmembrane domains, which is consistent with PafA fusion to the C-terminus of PD-L1 (Fig 2H).

### HSD17B12 negatively regulates PD-L1 in cancer cells independently of its catalytic activity

**Fig 3:**
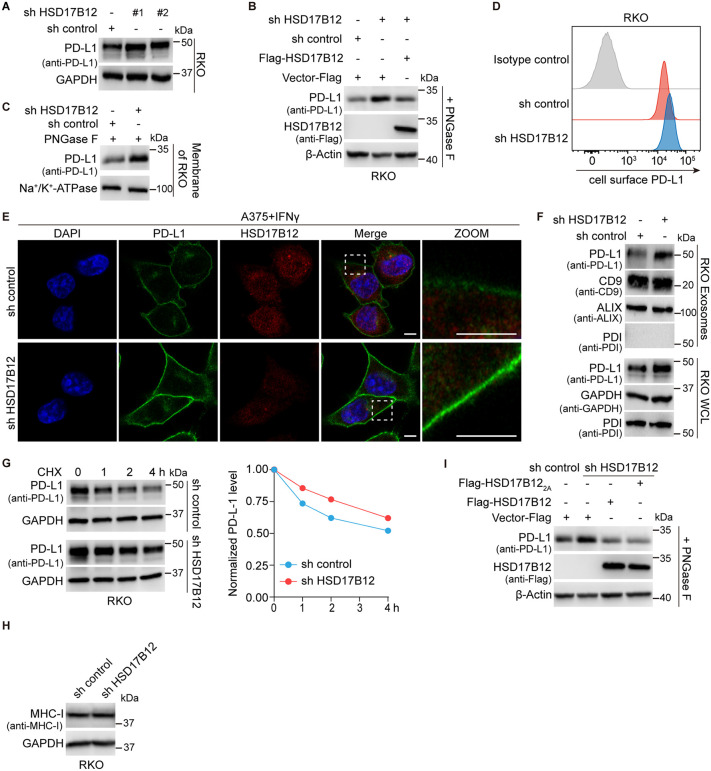
HSD17B12 negatively regulates PD-L1 independent of its catalytic activity. **(A)** HSD17B12 KD significantly increases PD-L1 expression in RKO cells. The GAPDH level was detected as a loading control. The experiment was performed three times. **(B)** Immunoblotting results showing HSD17B12 expression rescues enhanced PD-L1 expression in HSD17B12 KD RKO cells. This experiment was repeated three times independently with similar results. **(C–E)** HSD17B12 deficiency resulted in increased plasma membrane-located PD-L1 levels. RKO cells and HSD17B12 KD RKO cells were collected for immunoblotting (C) and flow cytometry (D). Experiments in C and D were repeated three times independently with similar results. (E) Immunofluorescence staining was performed using anti-PD-L1 and anti-HSD17B12 antibodies. Representative images are presented (scale bars: 5 μm). Three biological replicates. **(F)** Exosomal PD-L1 level significantly increases in HSD17B12 KD RKO cells. This experiment was repeated three times independently with similar results. **(G)** Stability of PD-L1 increased in HSD17B12 KD RKO cells. Cells were treated with 100 μg/mL CHX. The quantification is shown on the right. This experiment was repeated three times independently with similar results. **(H)** HSD17B12 deficiency does not affect MHC-I expression in RKO cells. Three biological replicates. **(I)** HSD17B12 regulates PD-L1 independent of its reductase activity. Representative of three experiments. Numerical data of (D) and (G) can be found in S1 Data, sheet “Fig 3”.

To investigate the impact of HSD17B12 on PD-L1, we designed distinct shRNAs to knockdown HSD17B12 in various human cancer cell lines (see Materials and methods for shRNA sequences). Quantitative real-time PCR (RT-qPCR) and immunoblotting confirmed the efficient depletion of HSD17B12 (S2A, S2B Fig). Knockdown of HSD17B12 led to a significant increase in PD-L1 expression in RKO cells (Fig 3A) as well as in HCT 116, SK-MEL-28, HeLa, and MDA-MB-231 cells (S2B–S2D Fig). Additionally, we found that HSD17B12 depletion further amplified IFN-γ-induced PD-L1 expression in RKO cells, A375 cells, and WM266−4 cells (S2E–S2G Fig). Restoring HSD17B12 expression in HSD17B12-deficient RKO cells reversed the increase in PD-L1 levels (Fig 3B). Increased surface PD-L1 expression was confirmed in HSD17B12 knockdown cells, as shown by immunoblotting, flow cytometry, and immunofluorescence (Fig 3C–3E). Similar effects were also observed in A375 cells (S2H, S2I Fig). Moreover, exosomal PD-L1 (exoPD-L1), which exhibits robust and persistent suppression effects of the anti-tumor immune response, was significantly elevated in HSD17B12-deficient RKO cells (Fig 3F). To investigate the effect of HSD17B12 on PD-L1 stability, we treated HSD17B12-deficient RKO cells with cycloheximide (CHX) to inhibit protein synthesis. This treatment revealed a reduction in PD-L1 degradation in HSD17B12-deficient cells (Fig 3G). In contrast, MHC class I (MHC-I) protein levels, used as a control, remained unaffected in both RKO and A375 cells following HSD17B12 knockdown (Figs 3H, S2J), demonstrating the specificity of HSD17B12’s regulatory effects on PD-L1. These findings highlight HSD17B12 as a broad regulator of PD-L1 expression in cancer cells.

Although HSD17B12 is known for its 3-ketoacyl-CoA reductase activity, the catalytically inactive HSD17B12-Y201A-K205A mutant (mutate Y201 and K205 residues to Ala) [32] retained its ability to regulate PD-L1 expression (Fig 3I). This finding suggests that HSD17B12 inhibits PD-L1 stability through mechanisms independent of its enzymatic activity.

### HSD17B12 promotes PD-L1 degradation via a lysosomal-dependent pathway

**Fig 4:**
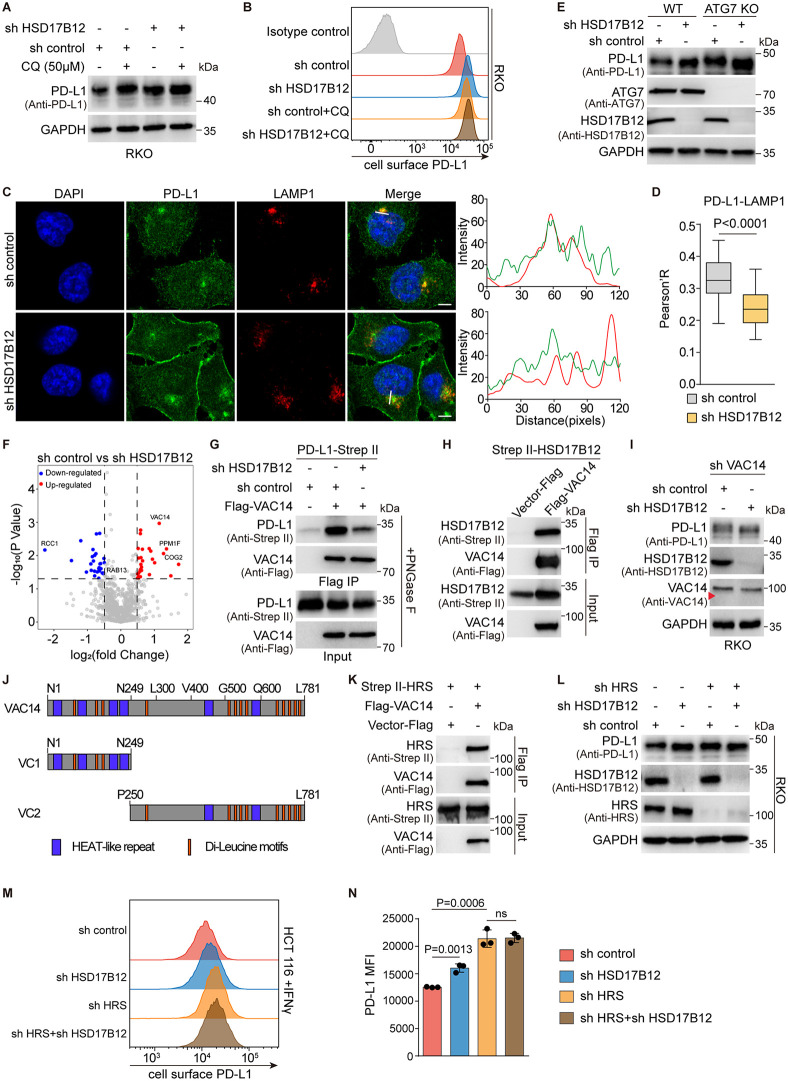
HSD17B12-mediated PD-L1 lysosomal degradation in a VAC14-ESCRT-dependent manner. **(A** and **B)** HSD17B12 regulates PD-L1 in a lysosome-dependent manner. RKO cells and HSD17B12 KD RKO cells were treated with 50 μM chloroquine for 24 hours and analyzed using immunoblot (A) and flow cytometry (B). Experiments in A and B were repeated three times independently with similar results. **(C** and **D)** HSD17B12 deficiency significantly reduces PD-L1 distribution to the lysosome. (C) Representative images are shown (scale bars: 5 μm). The intensity profiles of PD-L1 (green) and LAMP1 (red) along the white line are displayed in the right panel. Antibodies against PD-L1 and LAMP1 were used for staining. (D) The statistical analysis of the colocalization factor (Pearson’s R value). The statistics were plotted as mean ± SD and compared using a two-tailed Student *t* test. **(E)** HSD17B12 regulates PD-L1 independent of autophagy in RKO cells. Representative of three experiments. **(F)** Mass spectrometry (IP-MS) was employed to identify changes in the PD-L1 interactome upon HSD17B12 knockdown. The volcano plot depicts PD-L1-interacting proteins that were significantly altered by HSD17B12 KD (*p* value < 0.05, fold change > 1.4). **(G)** HSD17B12 knockdown dramatically reduces PD-L1 interaction with VAC14 in RKO cells. Immunoblots for input and IP were shown. This experiment was repeated three times independently with similar results. **(H)** VAC14 immunoprecipitates HSD17B12 in live cells. Three biological replicates. **(I)** HSD17B12’s effect on PD-L1 in RKO cells depended on VAC14. The experiment was performed three times. **(J)** The structure domains of VAC14. **(K)** VAC14 interacts with HRS in cells. Immunoblots for input and IP were shown. The experiment was performed three times. **(L–N)** HSD17B12 modulates PD-L1 in an HRS-dependent manner. Experiments in L and M were repeated three times independently with similar results. (N) Statistical analysis of PD-L1 mean fluorescence intensity (MFI). Values are mean ± SD from three independent experiments (*n* = 3). Statistical differences were determined by a two-tailed Student *t* tes*t*. Numerical data of (B), (C), (D), (F), (M), and (N) can be found in S1 Data, sheet “Fig 4”.

To clarify how HSD17B12 influences PD-L1 homeostasis, we treated cells individually with a proteasome inhibitor or a lysosome inhibitor. Treatment with the proteasome inhibitor MG132 further elevated PD-L1 expression in HSD17B12-deficient HCT 116 cells (S3A Fig). Consistently, the ubiquitination level of PD-L1 was not obviously altered by HSD17B12 knockdown (S3B Fig). In contrast, treating with the lysosome inhibitor chloroquine (CQ) did not further affect PD-L1 expression in HSD17B12 KD RKO cells and HSD17B12 KD WM266−4 cells (Figs 4A, S3C). Flow cytometry confirmed that CQ treatment did not enhance plasma membrane-localized PD-L1 in HSD17B12 KD RKO cells (Fig 4B), indicating that HSD17B12 likely regulates PD-L1 in a lysosome-dependent manner. Consistently, HSD17B12 depletion significantly reduced PD-L1 distribution to the lysosome (Fig 4C,4D) without affecting its distribution to the late endosome or recycling endosome in HSD17B12 KD A375 cells (S3D–S3G Fig). We further assessed whether HSD17B12 regulation of PD-L1 is dependent on the autophagy pathway. The ATG7-defective RKO clones, which block autophagy, were generated using the CRISPR-Cas9 system (S3H Fig). In these ATG7 knockout (KO) RKO cells, HSD17B12 deficiency still led to increased PD-L1 expression (Fig 4E), indicating that HSD17B12 promotes PD-L1 degradation independent of autophagy. Finally, to investigate whether HSD17B12 affects the biogenesis or dynamics of lysosomes in living cells, LysoTracker was employed to label acidic lysosomes. Immunofluorescence analysis demonstrated that HSD17B12 does not affect lysosome morphology within cells (S3I Fig). These results together demonstrate that HSD17B12 promotes PD-L1 degradation via a lysosome-dependent mechanism.

### HSD17B12-mediated PD-L1 degradation via a VAC14-ESCRT-dependent pathway

The balance between recycling and lysosomal degradation is central to the regulation of PD-L1. Proteins such as CMTM6 and HIP1R influence PD-L1 recycling and lysosomal degradation. We thus examined whether HSD17B12 interacts with these mechanisms [17,26,28]. HSD17B12 KD did not significantly impact PD-L1’s interactions with either HIP1R or CMTM6 (S4A, S4B Fig). In addition, there were no noticeable changes in the colocalization and interaction between PD-L1 and HSD17B12 in CMTM6 KO RKO cells (S4C–S4E Fig). In the CMTM6 KO context, knockdown of HSD17B12 still led to an increase in PD-L1 expression (S4F, S4G Fig). These results indicate that HSD17B12-mediated PD-L1 regulation is independent of CMTM6.

To further explore how HSD17B12 regulates PD-L1 degradation, we compared the interacting proteome of PD-L1 in control and HSD17B12 KD RKO cells. The proteins whose interactions with PD-L1 are dramatically altered by HSD17B12 deficiency were selected as potential regulators (Figs 4F, S4H). Co-immunoprecipitation confirmed that the interaction of PD-L1 with VAC14, but not with PPM1F, COG2, and RAB13, was dramatically attenuated in HSD17B12 KD RKO cells (Figs 4G, S4I–S4K). In addition, we demonstrated that VAC14 immunoprecipitates with HSD17B12 in living cells (Fig 4H). Knockdown of HSD17B12 did not affect PD-L1 expression in VAC14 KD RKO cells (Figs 4I, S4L). Together, these results suggested that VAC14 plays an important role in HSD17B12-mediated PD-L1 degradation.

Human VAC14 contains six HEAT-like repeats and multiple “dileucine pairs” (LeuLeu or LeuVal) (Fig 4J). HEAT-like repeats have a common function in mediating protein-protein interactions and have been reported to be present in numerous membrane trafficking proteins, such as VPS15 and huntingtin [33,34]. Yeast VAC14 (yVAC14) has been shown to control protein sorting into multivesicular-bodies (MVBs) [35], which delivers cargos to the vacuoles or lysosomes for degradation. The endosomal sorting complexes required for transport (ESCRT) are key mediators of MVB biogenesis. We demonstrated that VAC14 indeed interacts with hepatocyte growth factor-regulated tyrosine kinase substrate (HRS), a key component of ESCRT-0 [36], in cells (Fig 4K). To further confirm the requirement of ESCRT in HSD17B12-VAC14-induced PD-L1 degradation, we knocked down HRS and VPS28, a subunit of ESCRT-I, in cancer cells (S4M, S4N Fig). Silencing of HRS or VPS28 both stabilized PD-L1 in cancer cells, though further stabilization was not observed in HSD17B12-deficient cells (Figs 4L–4N, S4O). These findings suggest that HSD17B12 delivers PD-L1 to the lysosome in a VAC14-ESCRT-dependent manner.

### Cys272 is critical for the PD-L1-HSD17B12 interaction

Our PUP-IT experiments suggested that the cytoplasmic domain of PD-L1 interacts with HSD17B12 (Fig 2G). Emerging evidence has demonstrated that the cytoplasmic domain of PD-L1 plays important roles in regulating its stability and function via multiple pathways [11,14,22,23,27,37–42]. In particular, the regions “_265_RMLDVEKC_272_”, “_276_DTSSK_280_”, and “_286_QFEET_290_” in the mouse PD-L1 cytoplasmic domain have been identified as conserved motifs with potential regulatory roles [37]. We thus generated human PD-L1 mutants, lacking each of these conserved motifs (S5A Fig). Immunoprecipitation assays revealed a marked reduction in interaction between HSD17B12 and the PD-L1_△__R265-C272_ mutant (S5B Fig). Notably, Cys272, located within the “_265_RMLDVEKC_272_” motif, has been identified as a palmitoylation site that regulates PD-L1 stability by preventing its lysosomal degradation [27]. To test the significance of this residue, we mutated Cys272 to Ala (PD-L1_C272A_). The Cys272Ala mutation significantly weakened the interaction between PD-L1 and HSD17B12 (S5C Fig), confirming that Cys272 is a critical residue for the HSD17B12-PD-L1 interaction.

### Cys272 palmitoylation is not essential for HSD17B12-mediated PD-L1 degradation

To assess whether the palmitoylation of PD-L1 at Cys272 plays a role in HSD17B12-mediated PD-L1 degradation, we investigated the impact of HSD17B12 on PD-L1 palmitoylation and monoubiquitination [27]. Our analysis revealed that neither the palmitoylation nor the monoubiquitination levels of PD-L1 were significantly altered in HSD17B12-deficient RKO cells (S5D–S5E Fig). As previously reported, we observed that the C272A mutation, which inhibits palmitoylation, led to decreased PD-L1 expression [14,27] (S5F Fig). However, HSD17B12 KD still resulted in increased PD-L1_C272A_ expression (S5F Fig), suggesting that palmitoylation is not essential for HSD17B12’s effect on PD-L1 regulation. Consistently, PD-L1 levels in RKO cells treated with 2-bromopalmitate (2-BP), a general palmitoylation inhibitor, were also restored following HSD17B12 knockdown (S5G Fig). Furthermore, the stability of PD-L1-Ub, which fused a single ubiquitin to the intracellular domain of PD-L1 to mimic monoubiquitination, was also partially recovered by HSD17B12 knockdown in RKO cells (S5H Fig). Taken together, these findings suggest that Cys272 palmitoylation is not essential for HSD17B12-mediated PD-L1 regulation.

### HSD17B12 affects PD-1 binding and T cell-mediated cancer cell toxicity

**Fig 5:**
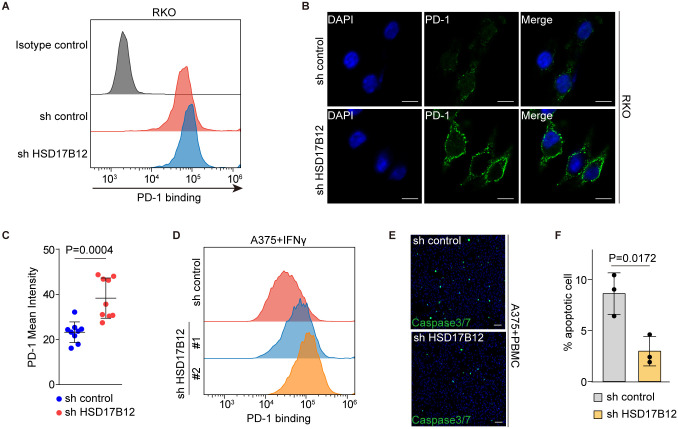
HSD17B12 modulates PD-1 binding and T cell-dependent cancer cell toxicity. **(A)** Flow cytometry results reveal enhanced PD-1 binding on HSD17B12 KD RKO cells. Three biological replicates. **(B** and **C)** Immunostaining showing stronger PD-1 binding on HSD17B12 KD RKO cells than control RKO cells. (B) Representative immunostaining results are shown (scale bars: 5 μm). (C) Statistical analysis of PD-1 intensity. The statistics were plotted as mean ± SD and compared using a two-tailed Student *t* test. **(D)** Flow cytometry indicates that PD-1 binding on HSD17B12 KD A375 cells increased, compared with A375 cells. Cells were treated with 10 ng/mL IFNγ for 48 hours prior to flow cytometry analysis. This experiment was repeated three times independently with similar results. **(E** and **F)** HSD17B12 KD reduces A375 cells’ sensitivity to T cell toxicity. Cell nuclei were labeled blue with Hoechst, and apoptotic cells were stained green with caspase-3/7 cleavage products. Scale bars: 100 μm. (F) Statistical analysis of three independent T cell killing assays. Values are mean ± SD from three independent assays (*n* = 3). Statistical differences were determined using a two-tailed Student *t* test. Numerical data of (A), (C), (D), and (F) can be found in S1 Data, sheet “Fig 5”.

Given PD-L1’s function in binding PD-1 to inhibit T cell activity, we examined whether HSD17B12 KD influences PD-1 interaction and T cell-mediated cancer cell killing. Using recombinant human PD-1 FC chimera protein, we observed that PD-1 binding was significantly higher on HSD17B12 KD RKO cells compared to control RKO cells, as shown by FACS analysis (Fig 5A). Immunofluorescence staining further confirmed the enhanced PD-1 binding on HSD17B12 KD RKO cells (Fig 5B, 5C). Enhanced PD-1 binding was also observed in IFN-γ-treated HSD17B12 KD A375 cells (Fig 5D). Furthermore, in T cell killing assays, HSD17B12 KD reduced A375 cells’ sensitivity to activated human peripheral blood mononuclear cells (PBMCs), indicating decreased T cell-mediated cytotoxicity (Fig 5E,5F). These results indicate that HSD17B12 affects PD-1 binding and T cell toxicity via regulating PD-L1 expression and highlight the potential of HSD17B12-mimetic drugs as promising candidates in cancer therapy.

### HSD17B12 mimic peptide promotes PD-L1 degradation and reduces tumor growth

**Fig 6:**
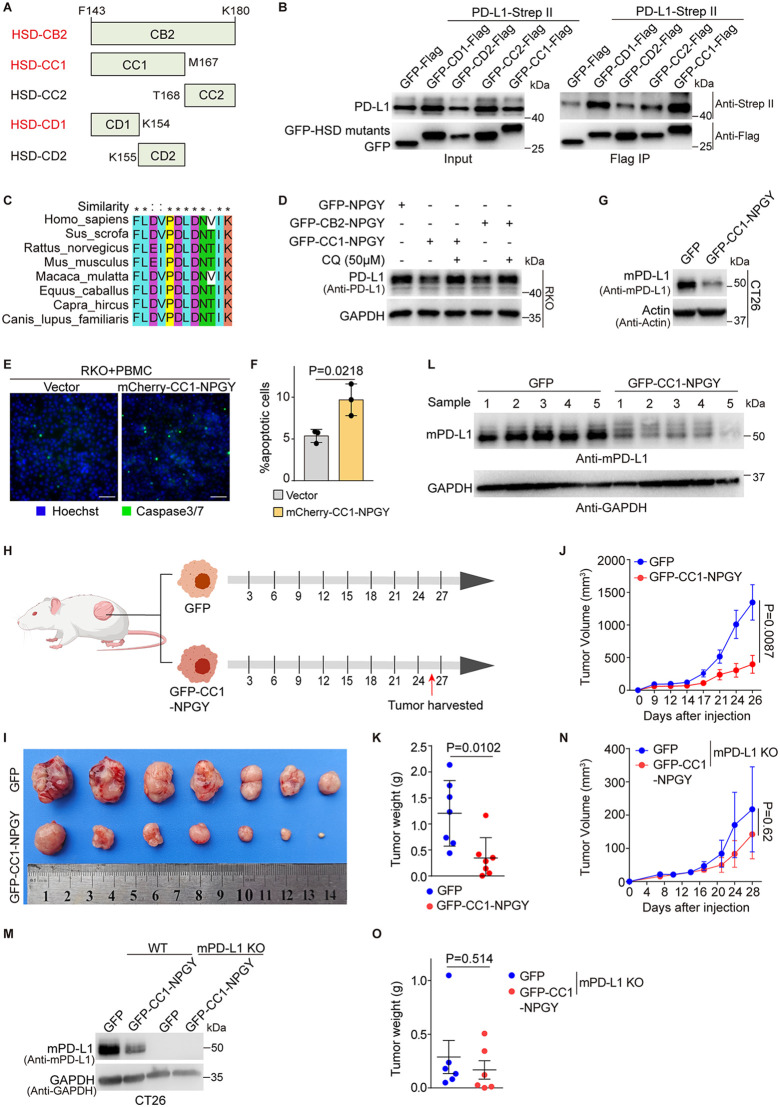
HSD17B12 mimic peptide stimulates PD-L1 degradation and inhibits tumor growth. **(A)** Constructs for mapping the PD-L1-binding domain in HSD17B12. **(B)** Immunoblotting results demonstrate that HSD-CC1 and HSD-CD1 interact with PD-L1. Immunoblots for input and IP were shown. The experiment was performed three times. **(C)** Sequence alignment indicating the conservation of F143-K154 in mammalian species. **(D)** HSD17B12 mimic peptides suppress PD-L1 expression via the lysosome. The GAPDH level was detected as a loading control. The experiment was performed three times. **(E** and **F)** HSD17B12 mimic peptide boosts T cell toxicity against RKO cells. (E) Cell nuclei were stained blue with Hoechst, whereas apoptotic cells were stained green with caspase-3/7 cleavage products. Representative images are shown (scale bars: 50 μm). (F) Statistical evaluation of the immunostaining images (E). The values are mean ± SD from three independent assays (*n* = 3). The statistical differences were determined using a two-tailed Student *t* test. **(G)** PD-L1 level significantly reduces in CT26 cells stably expressing the CC1-NPGY peptide. Representative of three experiments. **(H)** Schematic representation of the xenograft mouse model (created with BioGDP.com [43]). BALB/c mice were subcutaneously inoculated with CT26 cells stably expressing either GFP or GFP-CC1-NPGY. **(I–K)** CC1-NPGY suppresses CT26 tumor growth in mice as evidenced by tumor images (I), statistical analysis of tumor volumes (J), and tumor weight (K) from different groups (*n* = 7 per group). Values indicate mean ± SEM in (J) and mean ± SD in (K), compared by a two-tailed Student *t* test. **(L)** The CC1-NPGY peptide reduces PD-L1 expression levels in collected tumor cells. **(M)** Knockout of mPD-L1 in CT26 cells was confirmed by immunoblot analysis. The experiment was repeated three times. **(N-O)** CC1-NPGY lost its tumor-suppressive efficacy in the absence of PD-L1 as evidenced by statistical analysis of tumor volumes (N) and tumor weight (O) from different groups (*n* = 6 per group). Values indicate mean ± SEM in (N) and (O), compared by a two-tailed Student *t* test. Numerical data of (F), (J), (K), (N), and (O) can be found in S1 Data, sheet “Fig 6”.

The PUP-IT analysis revealed that Lys83 of HSD17B12, located in the cytoplasm between two transmembrane domains (C-1 region) (Fig 2G), is in proximity to PD-L1. Mutational studies identified the R24-K180 region (HSD-C1) as the important interacting domain for PD-L1 (S6A, S6B Fig). We therefore constructed a series of HSD17B12 mutants with deletions in various fragments of the C-1 region (Figs 6A, S6A). Among these, the K98-K180 region (HSD-CA2), but not the R24-F97 (HSD-CA1) of HSD17B12, was found to specifically interact with PD-L1 (S6C Fig). Further studies pinpointed the F143-K180 region (HSD-CB2) as critical for PD-L1 binding (S6A, S6D Fig), with the F143-M167 (HSD-CC1) fragment being specifically involved (Fig 6A,6B). The conserved F143-K154 sequence (HSD-CD1) was identified as the minimal effective binding motif (Fig 6A–6C).

Based on these findings, we designed peptides combining PD-L1 binding motifs with lysosomal targeting signals (NPGY, YKEL, and DQRDLI) to promote PD-L1 degradation (S6E Fig). Control peptides without lysosomal signals did not impact PD-L1 expression, whereas peptides with lysosomal signals significantly reduced PD-L1 in RKO cells (Figs 6D, S6F, S6G). Lysosomal inhibition via CQ treatment blocked PD-L1 degradation induced by HSD-CB2-NPGY and HSD-CC1-NPGY, confirming the involvement of lysosomes in this process (Fig 6D). The CHX assay confirmed that HSD-CC1-NPGY accelerated PD-L1 degradation (S6H Fig). Consistently, HSD-CC1-NPGY increased the sensitivity of RKO cells to activated T cells (Fig 6E, 6F). These findings suggested that HSD17B12 mimic peptides are promising candidates to stimulate PD-L1 degradation and enhance T cell-mediated toxicity.

Encouraged by these findings, we next examined the anti-tumor activity of HSD-CC1-NPGY in mouse models. We first confirmed that HSD17B12 negatively regulates PD-L1 expression in CT26 murine colon cancer cells (S6I Fig). Subsequently, CT26 cells were infected with lentivirus expressing either GFP (control) or GFP-CC1-NPGY. Western blot analysis confirmed that GFP-CC1-NPGY significantly reduced both the expression of PD-L1 and exoPD-L1 levels in CT26 cells (Figs 6G, S6J). These stable cell lines were then subcutaneously implanted into BALB/c mice for in vivo evaluation (Fig 6H). Notably, HSD-CC1-NPGY suppresses CT26 tumor growth in immunocompetent mice but not in immunodeficient mice (Figs 6I–6K, S6K–S6M). Tumor analysis confirmed a marked reduction in PD-L1 levels (Fig 6L). Furthermore, to determine whether the tumor-suppressive effect of CC1-NPGY in vivo is specifically mediated through PD-L1 downregulation—rather than an immune response against the peptide itself—we generated a PD-L1 KO CT26 cell line using the CRISPR/Cas9 system. Successful ablation of murine PD-L1 was validated by western blot analysis (Fig 6M). Notably, CC1-NPGY no longer exerted inhibitory effect on tumor growth (Figs 6N, 6O, S6N), indicating that the in vivo anti-tumor activity of CC1-NPGY depends on PD-L1.

Collectively, these findings demonstrate that the HSD17B12-derived peptide effectively down-regulates PD-L1 and suppresses tumor progression in vivo, suggesting the potential of HSD17B12 as a novel therapeutic target for cancer.

## Discussion and conclusion

The low efficiency of cancer immunotherapy and tumor heterogeneity highlight the urgent need for innovative target and combined therapy strategies. This study identifies HSD17B12 as a modulator of immune responses and highlights its potential as a therapeutic target in cancer treatment. HSD17B12 promotes the lysosomal degradation of PD-L1 via a VAC14-ESCRT-dependent pathway, thereby facilitating cytotoxic T cell infiltration, particularly in CRC. Moreover, a synthetic peptide mimicking HSD17B12’s function effectively induced T cell-mediated cytotoxicity and suppressed tumor growth in vivo, emphasizing the potential of HSD17B12 as a therapeutic target.

We employed the PUP-IT proximity labeling system [30] to identify PD-L1-interacting proteins, including HSD17B12, and verified their interaction using CO-IP. To rule out the possibility that interaction/labeling happens after lysis, we also performed a post-lysis proximity labeling experiment. Specifically, PD-L1-PafA and RKO-HSD17B12-Flag (KI) were separately expressed in RKO cells, and no detectable labeling on HSD17B12 was observed in the mixed lysates, which demonstrates that the PD-L1–HSD17B12 interaction occurs authentically in living cells and is not an artifact of the experimental lysis procedure.

The stability of PD-L1 is regulated by multiple pathways. Previous studies have demonstrated that CMTM6/4 inhibits lysosomal delivery of PD-L1, while HIP1R promotes it in a ubiquitin-independent manner. Additionally, palmitoylation of PD-L1 at C272 suppresses its monoubiquitination, preventing its ESCRT-mediated lysosomal trafficking. In contrast, this study identifies HSD17B12 as a novel regulator of PD-L1, acting independently of CMTM6/4, HIP1R, and palmitoylation. Our preliminary analysis of CRC patient samples suggested a correlation between PD-L1 and HSD17B12 expression, highlighting HSD17B12 as a promising target for cancer therapies.

Tumorigenesis, a process of clonal evolution, causes complex genetic and molecular alterations that vary widely across cancers. The functional and phenotypic heterogeneity observed in tumors underscores the necessity for precision therapies targeting specific pathways. Our findings highlight the potential of leveraging HSD17B12-based therapies to restore immune surveillance. This strategy exemplifies how mechanistic insights lead to innovative and tailored therapeutic targets for cancer.

Clinically approved anti-PD-L1 therapies target the extracellular domain of cell-surface PD-L1. However, intracellular stores of PD-L1, which are dynamically trafficked to the cell membrane and exosomes, often limit therapeutic effectiveness. Both cell-surface PD-L1 and exoPD-L1 play crucial roles in immunosuppression, tumor progression, and therapy resistance [44–50]. Recent studies have shown that removing exoPD-L1 can inhibit tumor growth even in anti-PD-L1-resistant mouse models, emphasizing the need for comprehensive targeting of PD-L1. Our results demonstrate that HSD17B12 modulates the degradation of both whole-cell PD-L1 and exoPD-L1, providing opportunities for comprehensive targeting. We developed a chimeric peptide combining HSD17B12’s PD-L1-binding motifs with lysosomal sorting signals. This peptide successfully promoted lysosomal degradation of PD-L1, reducing tumor growth in vivo. Future efforts to develop cell-permeable versions of these peptides could further enhance their therapeutic potential.

Importantly, the HSD17B12-derived peptide demonstrated efficacy in suppressing tumor growth in CRC mouse models. The HSD17B12-derived peptide specifically targets the cytoplasmic domain of PD-L1 (PD-L1-CD), an area with low sequence similarity to other B7 family molecules, making it an attractive therapeutic target. This specificity also supports the peptide’s potential in combination therapies.

In summary, our study provides insight into HSD17B12’s involvement in enhancing anti-tumor immunity across a variety of malignancies. Both HSD17B12 and its derived peptide represent promising capacity to enhance T cell-mediated anti-tumor responses, offering a foundation for the development of cancer therapies by targeting HSD17B12.

## Materials and methods

### Ethics statement

The study was approved by the Research Ethics Committee of West China Hospital, Sichuan University (permission number: 2020 (374)) and was conducted in accordance with the ethical principles outlined in the Declaration of Helsinki. Informed consent was obtained from all patients prior to their participation, with thorough review and documentation. All animal-related research was approved by the Animal Ethics Committee of West China Hospital, Sichuan University (Approval Number: 20240301110).

### Cell culture and transfection

HEK293T cells (RRID: CVCL_0063), HeLa cells (RRID: CVCL_0030), WM266-4 cells (RRID: CVCL_2765), A375 cells (RRID: CVCL_0132), SK-MEL-28 cells (RRID: CVCL_0526), CT26 cells (RRID: CVCL_7254), and HCT 116 (RRID: CVCL_0291) cells were obtained from the National Collection of Authenticated Cell Cultures. RKO (RRID: CVCL_0504) cells and MDA-MB-231(RRID: CVCL_0062) cells were purchased from iCell Bioscience RKO cells were maintained in RPMI 1640 medium supplemented with 10% fetal bovine serum (FBS) and 100 U/ml penicillin-streptomycin. Other cells were maintained in Dulbecco’s modified Eagle’s medium (DMEM) supplemented with 10% FBS and 100 U/ml penicillin-streptomycin. All cells were cultured in a humidified incubator at 37 °C under 5% CO_2_. All cells were routinely tested negative for mycoplasma contamination. Transient transfection was performed using PEI or Lipofectamine 2000 according to the manufacturer’s instructions.

### Plasmids

The mammalian expression vectors for PD-L1 and CMTM6 were synthesized by GENERAL BIOSYSTEMS. HSD17B12, VAC14, HIP1R, RAB13, COG2, PPM1F, and HRS genes were amplified from the HEK293T or RKO cDNA library and cloned into the pCMV vector with designed tags. The sgRNA 5′-CTTGAAAGACTCGAGTGTGT-3′ was used to generate RKO ATG7 KO clones, and the sgRNA 5′-TCACAATGTACTTTATGTGG-3′ was used to generate RKO CMTM6 KO clones. The sgRNA 5′- GTATGGCAGCAACGTCACGA-3′ was used to generate CT26 mPD-L1 KO clones. PD-L1 and HSD17B12 point mutation mutants were generated by site-directed mutagenesis. The GFP chimeric proteins were generated by inserting the indicated HSD17B12 sequences into the pcDNA3.1-EGFP-Flag vector using homologous recombination and site-directed mutagenesis. All plasmids were sequenced to verify their correctness.

### Antibodies and reagents

The following antibodies were used for western blot analyses: anti-Flag (M20008, 1:2000, Abmart, RRID: AB_2713960), anti-HA (ab9110, 1:2000, Abcam, RRID: AB_307019), anti-Strep II (HA500061, 1:2000, HUABIO, RRID: AB_2904531), anti-GAPDH (AC002, 1:10000, ABclonal, RRID: AB_2736879), anti-Sodium Potassium ATPase (ab76020, 1:10000, Abcam, RRID: AB_1310695), anti-HSD17B12 (ab236990, 1:2000, Abcam, RRID: AB_2876866), anti-PD-L1 (13684, 1:2000, CST, RRID: AB_2687655), anti-PD-L1 (ab213480, 1:2000, Abcam, RRID: AB_2773715), anti-MHC-I (ET1702-47, 1:1000, HUABIO, RRID: AB_3070309), anti-ALIX (67715-1-Ig, 1:2000, Proteintech, RRID: AB_2882905), anti-CD9 (60232-1-Ig, 1:2000, Proteintech, RRID: AB_11232215), anti-CD9 (ET1601-9, 1:2000, HUABIO, RRID: AB_3069620), anti-PDI (WH319294, 1:1000, ABclonal), anti-ATG7 (T55658S, 1:2000, Abmart), anti-biotin (GTX124262-S, 1:5000, GeneTex, RRID: AB_11174491), Streptavidin-HRP (A0303, 1:2000, Beyotime), anti-Ub (sc-8017, 1:200, Santa Cruz Biotechnology, RRID: AB_628423), anti-CMTM6 (HPA026980, 1:1000, Sigma, RRID: AB_10602801), anti-HRS (PTG-10390, 1:2000, Proteintech, RRID: AB_2118914), anti-VPS28 (ab167172, 1:2000, Abcam), anti-VAC14 (PTG-15771, 1:1000, Proteintech, RRID: AB_2214753), HRP conjugated goat anti-rabbit IgG (H + L) (SA00001–2, 1:2000, Proteintech, RRID: AB_2722564), HRP conjugated goat anti-mouse IgG (H + L) (31,430, 1:2000, Thermofisher, RRID: AB_228307).

The following antibodies were used for flow cytometry analyses: APC anti-human CD274 (329708, 1:100, Biolegend, RRID: AB_940360) and APC mouse IgG2b (400322, 1:100, Biolegend, RRID: AB_326500).

The following antibodies were used for immunofluorescence staining: anti-PD-L1 (LS-C338364, 1:100, LSBio, RRID: AB_2941014), anti-LAMP1 (9091S, 1:100, CST, RRID: AB_2687579), anti-RAB11 (5589S, 1:50, CST), anti-RAB7B (ab137029, 1:100, Abcam, RRID: AB_2629474), anti-HSD17B12 (ab236990, 1:100, Abcam, RRID: AB_2876866), anti-CMTM6 (HPA026980, 1:100, Sigma, RRID: AB_10602801), anti-CD3 (ab237721, 1:4000, Abcam), anti-CD8 (ZA-0508, ZSGB-BIO, RRID: AB_2890107), anti-GZMB (ab255598, 1:8000, Abcam, RRID: AB_2860567), IgG H&L (HRP) (ab205718, 1:4000, Abcam, RRID: AB_2819160), goat anti-mouse highly cross-adsorbed Alexa Fluor Plus 488 (A32723, 1:500, Thermofisher, RRID: AB_2633275), goat anti-rabbit IgG (H + L) highly cross-adsorbed Alexa Fluor Plus 594 (A32740, 1:500, Thermofisher, RRID: AB_2762824), goat anti-rabbit IgG (H + L) highly cross-adsorbed Alexa Fluor Plus 647 (A32728, 1:500, Thermofisher, RRID: AB_2633277), goat anti-human IgG (H + L) highly cross-adsorbed Alexa Fluor Plus 488 (A11013, 1:500, Thermofisher, RRID: AB_141360).

The following antibodies were used for immunohistochemistry staining: anti-HSD17B12 (ab236990, 1:100, Abcam, RRID: AB_2876866).

MG-132 (S2619, Selleck, CAS: 1211877-36-9), chloroquine (HY-17589, MCE, CAS: 54-05-7), cycloheximide (S7418, Selleck, CAS:66-81-9), 2-BP (238422, Sigma, CAS: 18263-25-7), IFNγ (RP01038, ABclonal), Biotin-HPDP (APEXBIO, A8008, CAS: 129179-83-5), Recombinant Human PD-1 Fc Chimera Protein (1086-PD-050, R&D), visualized with iFluor 430 Tyramide (AAT Bioquest, 45096), iFluor 488 Tyramide (AAT Bioquest, 11060), CY3 Tyramide (AAT Bioquest, 11065), 4′,6-diamidino-2-phenylindole (DAPI) (Solarbio, C0060, CAS: 28718-90-3), NEM (04260, Sigma, CAS: 128-53-0), HAM (467804, Sigma, CAS:7803-49-8), Ultra DAB kit (DAB-4033, MXB Biotechnologies), hygromycin B (S160J7, Basalmedia, CAS: 31282-04-9), and puromycin (HY-B1743A, MCE, CAS: 58-58-2) were purchased from the indicated suppliers.

### Construction of knockdown cell lines

The shRNA sequences for HSD17B12 are: 5′-GGATAAACTTGACCAGGTTTC-3′ (#1); 5′-GCCTACCTAGCCCTGCGTAT-3′ (#2). The shRNA sequence for VAC14 is: 5′-TCCAGATCCTGGGTGACAATG-3′. The shRNA sequences for HRS are: 5′-CTCACGTCCGGAGTAACACTA-3′, and 5′-AGACAGATTGGGAGTCCATTT-3′. The shRNA sequences for VPS28 are: 5′-GAGGGAGAAGTACGACAACAT-3′ and 5′-GTCAGCCTACAACGCCTTCAA-3′ [51].

The lentiviral transfer vector and the packaging plasmid, psPAX2, and pMD2.G, were transfected into HEK293T cells. At 24 hours and 48 hours after transfection, the supernatant with virus was collected, filtered through a 0.45 μm filter. The virus was added to cells; puromycin or hygromycin was added to select cells that knocked down the gene of interest.

### Sample purification and mass spectrometry analysis

For the PUP-IT assay, HEK293T cells expressing PD-L1-Flag-PafA or Flag-PafA and Bio-Pup(E) were lysed in lysis buffer (50 mM HEPES, 1.5 mM NaCl, 1% TritonX-100, 1.5 mM MgCl_2_, 1 mM EGTA, 10% glycerol, pH = 7.5). Urea was added to the cell lysate to a final concentration of 8 M. Streptavidin beads were incubated with the cell lysate overnight at 4 °C. Then, the beads were sequentially washed twice with buffer 1 (50 mM Tris pH = 8.0, 8 M urea, 200 mM NaCl, 0.2% SDS) and buffer 2 (50 mM Tris pH = 8.0, 8 M urea, 200 mM NaCl, 2% SDS), followed by washing twice with buffer 3 (50 mM Tris pH = 8.0, 8 M urea, 200 mM NaCl) and buffer 4 (50 mM Tris pH = 8.0, 0.5 mM EDTA, 1 mM DTT). Finally, the samples were washed three times with 50 mM NH_4_HCO_3_ aqueous solution at room temperature. Resuspend the beads with 50 mM ammonium carboxylate and perform on-bead trypsin digestion. The peptides were analyzed using liquid chromatography-tandem mass spectrometry (HPLC-MS/MS, Ultimate 3000 system, ThermoFisher Scientific, USA, RRID: SCR_026145) by Bio-Tech Pack Technology Company (Beijing). The raw data were processed and searched with MaxQuant 1.5.4.1 (RRID: SCR_014485). Two missed trypsin cleavages were allowed, and cysteine carbamidomethylation was set as a fixed modification. GGE (K), oxidation (M), acetyl (protein N-terminus), and deamidation (NQ) were set as variable modifications.

To identify changes in the PD-L1 interactome following HSD17B12 knockdown, mass spectrometry (IP-MS) was performed. RKO sh control cells and sh HSD17B12 cells expressing PD-L1-Strep II were harvested for mass spectrometry analysis. The cells were lysed in lysis buffer (50 mM Tris-HCl pH = 8.0, 150 mM NaCl, 5 mM EDTA, and 0.5% NP-40) supplemented with protease inhibitor cocktail for 30 min on ice. Strep II tag beads were incubated with the cell lysate for 4 hours at 4 °C. Then, the beads were washed with lysis buffer four times, once for 5 min at 4 °C. SDS loading buffer was added to the Strep II beads and heated at 95 °C for 10 min. The samples were run on SDS-PAGE gels, and the gels with samples were cut and collected carefully. The purified samples were digested with trypsin overnight at 37 °C and analyzed using liquid chromatography-tandem mass spectrometry (HPLC-MS/MS, Exploris 480, ThermoFisher Scientific, USA, RRID: SCR_022215).

### Immunofluorescence (IF) staining of cells

The cells were washed three times with PBS, then fixed and permeabilized with cold methyl alcohol for 5 min. Samples were blocked with 5% BSA at room temperature for 1 hour and incubated with primary antibodies overnight at 4 °C. Secondary antibodies were incubated at room temperature for 1 hour, followed by washing three times with PBS. Coverslips were mounted onto slides with a mounting medium containing DAPI. The confocal laser scanning microscope (ZEISS LSM 880, RRID: SCR_020925) was used to acquire the images. Colocalization analysis was performed using Image J (RRID: SCR_003070).

### Immunoprecipitation

Cells were collected 48 hours after transfection and washed three times with PBS. Lyse the cells in lysis buffer (50 mM Tris-HCl pH = 8.0, 150 mM NaCl, 5 mM EDTA, and 0.5% NP-40) supplemented with protease inhibitor cocktail for 30 min on ice. The cell lysates were centrifuged (15,000 × *g,* 15 min) at 4 °C and the supernatant was collected. Anti-Flag affinity beads or anti-Strep II affinity beads were added to the lysate and incubated for 4–6 hours at 4 °C with constant rotation. The beads were washed three times with lysis buffer and denatured in SDS loading buffer at 95 °C for western blotting.

### Reverse Transcription Quantitative real-time PCR (RT-qPCR) analysis

Total RNA was isolated using a Cell Total RNA Isolation Kit (FOREGENE). For each sample, 1 μg of total RNA was reverse transcribed into cDNA using HiScript II Q RT SuperMix (#R223-01, Vazyme). RT-qPCR was then performed with AceQ Universal SYBR qPCR Master Mix (#Q511-02, Vazyme) on a Bio-Rad CFX96 PCR System (RRID: SCR_018064). Amplification of GAPDH was used as an internal control. Quantification was calculated using the 2^−∆∆CT^ method and displayed as fold change. The following primer sets were used: 5-CCAGGTGGTCTCCTCTGACTT-3 and 5-GTTGCTGTAGCCAAATTCGTTGT-3 for GAPDH; 5-AGCAAAGCATGGAATGAAGGTTGT-3 and 5-CTAAGATGCCGATTTCAAGACCAGC-3 for HSD17B12.

### Exosomes isolation

Cell culture media was centrifuged at 300 × *g* for 10 min at 4 °C, followed by 2,000 × *g* for 10 min at 4 °C and 10,000 × *g* for 30 min at 4 °C. The supernatant was centrifuged at 100,000 × *g* for 70 min at 4 °C. Wash the pellets with cold PBS, followed by centrifuging at 100,000 × *g* for 70 min at 4 °C. Finally, the pellets were denatured in SDS loading buffer at 95 °C for western blotting.

### Acyl-Biotin Exchange (ABE) assay

Cells expressing PD-L1-Flag were lysed in lysis buffer (50 mM Tris-HCl, 150 mM NaCl, 1 mM MgCl_2_, 1% NP-40, 10% glycerol, and 50 mM N-Ethylmaleimide) (pH = 7.5) for 1.5 hours at 4 °C. The lysates were centrifuged (15,000 × *g*, 15 min) at 4 °C. The supernatants were then incubated with anti-Flag beads at 4 °C for 4 hours. Wash the beads five times with lysis buffer (pH = 7.5), followed by three times washing with lysis buffer (pH = 7.2). The beads were then incubated with a freshly prepared hydroxylamine (HAM)-containing lysis buffer (50 mM Tris-HCl, 150 mM NaCl, 1 mM MgCl_2_, 1% NP-40, 10% glycerol, and 1 M HAM, pH = 7.2) at room temperature for 1 hour. Wash the beads four times with lysis buffer (pH = 7.2) and three times with lysis buffer (pH = 6.2). The beads were treated with Biotin-HPDP (5 μM) in lysis buffer (pH = 6.2) at 4 °C for 1 hour and analyzed by western blotting.

### Detection of cell-surface PD-L1

Collected cells were resuspended in 200 μL cell staining buffer (#420201, BioLegend) or PBS and incubated with APC-conjugated anti-human PD-L1 antibody at room temperature for 30 min. The samples were washed three times with PBS and analyzed by flow cytometry. Cells in FSC-H (1 × 10^6^ − 5 × 10^6^) and SSC-H (2 × 10^5^ − 5 × 10^5^) were defined as “positive” cells and gated for PD-L1 APC channel analysis.

To isolate the plasma membrane proteins, HDB buffer (140 mM NaCl, 5 mM KCl, 12.5 mM HEPES, 0.5 mM EDTA, 5 mM MgCl_2_, pH = 7.4) supplemented with a protease inhibitor cocktail was added to the cells. After repeating freeze-thaw cycles five times, samples were centrifuged (12,000 × *g*, 10 min) at 4 °C, and the supernatant was decanted. Sediment was resuspended with membrane extraction buffer (1.5 mM NaCl, 50 mM HEPES, 1.5 mM MgCl_2_, 1 mM EGTA, 10% glycerol, 1% Triton X-100, pH = 7.5) and rotated at 4 °C for 30 min, followed by centrifugation (15,000 × *g*, 15 min) at 4 °C. Then the supernatant was denatured by SDS loading buffer at 70 °C for western blotting.

### Deglycosylation of PD-L1

PD-L1 was deglycosylated using PNGase F (#P0704L, New England Biolabs) according to the manufacturer’s protocol. Briefly, cells were lysed using RIPA or IP buffer (50 mM Tris-HCl pH = 8.0, 150 mM NaCl, 5 mM EDTA, and 0.5% NP-40), and the lysates were centrifuged at 15,000 × *g* for 15 min at 4 °C. Supernatant (30 μL) was mixed with Glycoprotein Denaturing Buffer (3.3 μL) and denatured at 95 °C for 10 min. The samples were then incubated with 4 μL of 10% NP-40, 4 μL of GlycoBuffer 2, and 0.6 μL of PNGase F at 37 °C for 2–3 hours, followed by denaturation at 95 °C in loading buffer.

### Post-lysis proximity labeling

RKO-WT cells and RKO HSD17B12-Flag knock-in cell lines were transfected with PD-L1-Flag-PafA and Bio-Pup(E) plasmids. Six hours after transfection, 5 μM biotin (#B4639, Sigma) was added followed by continued culture for 48 hours. RKO-WT cells expressing PD-L1-Flag-PafA, RKO HSD17B12-Flag knock-in cells expressing PD-L1-Flag-PafA, and RKO HSD17B12-Flag knock-in cells not expressing PD-L1-Flag-PafA were separately lysed using 500 μL Lysis Buffer (50 mM Tris-HCl pH = 8.0, 150 mM NaCl, 5 mM EDTA, and 1% NP-40). All three samples were lysed on ice for 30 min, then centrifuged at 15,000 × g for 15 min at 4 °C. Then the RKO-WT lysate was mixed with the RKO HSD17B12-Flag knock-in lysate not expressing PD-L1-Flag-PafA, while the RKO HSD17B12-Flag knock-in lysate expressing PD-L1-Flag-PafA was supplemented with an additional 500 μL Lysis Buffer. Subsequently, 15 μL of streptavidin beads were added to both samples and incubated at 4 °C for 3 hours. After centrifugation, the supernatant was collected and incubated with 10 μL Flag beads at 4 °C for another 3 hours. Both streptavidin and Flag beads were washed, resuspended in loading buffer, and denatured at 95 °C for western blotting.

### Western blotting

Cells were lysed in RIPA lysis buffer with a protease inhibitor cocktail for 30 min on ice. The cell lysates were centrifuged (15,000 × *g,* 10 min) at 4 °C, and the supernatant was collected, followed by denaturing. The samples were run on SDS-PAGE gels and transferred to PVDF membranes. The PVDF membranes were blocked with 5% milk in TBST for 2 hours at room temperature and incubated with corresponding primary antibodies overnight at 4 °C. The PVDF membranes were washed three times with TBST and incubated with HRP-conjugated secondary antibodies at room temperature for 2 hours. Finally, the western blot bands were detected by the ECL chemiluminescence system.

### PD-L1/PD-1-binding assay

Cells were collected and washed twice with PBS. The cells were resuspended in 200 μL cell staining buffer (#420201, BioLegend) and incubated with 5 μg/mL recombinant human PD-1 FC chimera protein (1:100) at room temperature for 30 min. The cells were washed three times with cell staining buffer and incubated with the anti-human Alexa Fluor 488 dye-conjugated antibody at room temperature for 30 min. The samples were washed three times with cell staining buffer and analyzed by flow cytometry. Cells in FSC-H (1 × 10^6^ − 4 × 10^6^) and SSC-H (2 × 10^5^ − 1 × 10^6^) were defined as “positive” cells and gated for FITC channel analysis.

### Animal experiments

Five-week-old female BALB/c and BALB/c-Nude mice were purchased from GemPharmatech Company (Nanjing, China) and quarantined for 1 week before inoculation of CT26 cells. These mice were housed at a density of up to 5 animals per cage with free access to water and food under a 12-hour light/dark cycle. CT26 cells (8 × 10^5^) stably expressing HSD-CC1-NPGY or GFP were suspended in 100 μL DMEM and injected subcutaneously into the flanks of the mice. The tumor volumes were measured every 2–4 days. Tumor volume (mm^3^) was calculated as π/6 × length × width^2^. The weight of the tumors was recorded on the day of sacrifice. For immunoblotting, tumors were collected and processed into single-cell suspensions by digestion in collagenase D (1 mg/mL, Roche) and DNase I (0.2 mg/mL, Roche) at 37 °C for 1 hour. After filtering with a 70 μm filter, the cells were lysed in RIPA lysis buffer with protease inhibitor cocktail on ice for 30 min. The cell lysates were centrifuged (15,000 × *g,* 10 min) at 4 °C, and the supernatant was collected. SDS loading buffer was added and boiled at 95 °C for 10 min.

### T cell cytotoxicity assay

Human peripheral blood mononuclear cells (PBMCs) were isolated from the peripheral blood of volunteers by density gradient centrifugation [31]. The PBMC cells were activated with 100 ng/mL CD3 antibody (RRID: AB_571924), 100 ng/mL CD28 antibody (RRID: AB_314315), and 10 ng/mL IL-2 for 48 hours. At the same time, A375 sh control cells and sh HSD17B12 cells were seeded in a 96-well plate at a density of 20,000 cells per well. The activated PBMC cells were incubated with A375 cells at a 5:1 ratio in the presence of fluorescence caspase-3/7 substrate in a 37 °C incubator for 12 hours. Cells were washed with PBS after co-incubation and incubated with Hoechst in a 37 °C incubator for 10 min. Cells were washed twice with PBS and observed under a fluorescence microscope.

### Biospecimen collection and clinical information

A total of 126 tumor tissue samples and 104 paired distant normal tissues from colorectal cancer (CRC) patients were collected at West China Hospital, Sichuan University. Demographic and clinical information, including gender, age, overall survival (OS) status, OS duration in months, tumor location, TNM stage, and mismatch repair (MMR) status (categorized as either deficient (dMMR) or proficient (pMMR), was collected and is detailed in S2 Table. Samples analyzed in Fig 1A–1G were obtained from treatment-naïve patients without any prior systemic or neoadjuvant therapy. The cohort for analyses in Fig 1H,1I comprised 7 patients with dMMR tumors who were treated with anti-PD-1 therapy—tislelizumab 200 mg IV every 3 weeks, sintilimab 200 mg IV every 3 weeks (one exploratory case 3 mg/kg every 2 weeks), or serplulimab 3 mg/kg (≈ 200 mg) IV every 2 weeks. Treatment was administered either as monotherapy or in combination with pelvic radiotherapy and cytotoxic chemotherapy (CapeOX or, in selected exploratory cases, modified FOLFOXIRI), providing a representative neoadjuvant-total-therapy context.

### Survival analysis

The R package “survminer” was utilized to determine the optimal cutoff point for HSD17B12 expression across the 104 tumor samples. Differences in survival between groups stratified by high and low HSD17B12 expression were assessed using the log-rank test. The survival curves were generated using the Kaplan–Meier method with the R package “survival” (RRID: SCR_021137) and visualized using the “survminer” package (RRID: SCR_021094). Detailed patients and tumor signatures can be found in S2 Table.

### Calculation of activated CD8^+^ T cell score

Enrichment scores for activated CD8^+^ T cells were calculated for each tumor sample using imputed proteomic data [52,53]. This was achieved through ssGSEA, which quantitatively evaluates the relative abundance of a predefined gene set in individual samples. The analysis was performed using the R package “GSVA” (RRID: SCR_021058). The gene set used for this analysis was curated from a previous study [54].

### Quantitative western blotting analysis

Western blot images were initially processed using ImageJ (RRID: SCR_003070) for background subtraction to remove nonspecific signals and enhance quantitative accuracy. The images were converted to grayscale to improve contrast and facilitate intensity analysis. Each band was outlined using the rectangular selection tool in ImageJ, and the grayscale intensity values were extracted for further analysis. To normalize the data, the intensity values of each band were first normalized to the loading control (GAPDH). To standardize results across multiple membranes, normalization coefficients were determined for each membrane using a common reference sample (T66). Specifically, the mean intensity of the common reference sample was calculated across all membranes, and the normalization coefficient for each membrane was obtained by dividing the raw intensity of the reference sample by this mean. The final normalized intensity values for each sample were calculated by dividing their GAPDH-normalized values by the corresponding normalization coefficient for the membrane on which the sample was analyzed. These normalization steps ensured consistency and comparability across different blots.

### Multiplexed IF staining and quantification of tumor tissues

Formalin-fixed, paraffin-embedded (FFPE) tumor tissues were sectioned into consecutive 5 μm slices. The slides were first deparaffinized using an eco-friendly deparaffinization agent, with three washes of 10 min each, followed by rehydration in graded ethanol solutions (absolute ethanol, 95% ethanol, and 75% ethanol, 5 min each). The slides were then rinsed three times with distilled water for 3 min each. Antigen retrieval was performed by placing the tissue sections in antigen retrieval buffer (pH 9.0 ethylenediaminetetraacetic acid) within a pressure cooker. The cooker was heated on an induction stove set to 2,100 watts until boiling, then the tissue sections were added. The lid was secured, and after the steam valve rose, the pressure regulator was applied (stove was set to 800−1,200 watts). Once steam began venting, the tissue sections were retrieved after precisely 1 min and 30 s of heating. The cooker was cooled under running water until pressure was released, and the slides were allowed to cool naturally in the retrieval solution to room temperature (25−30 °C, ~1 hour). The slides were then removed and washed in TBS for 5 min, three times. To block endogenous peroxidase activity, the slides were incubated in 3% H_2_O_2_ for 20 min at room temperature, protected from light. TSA staining was then performed for the following markers: CD3 (Abcam, ab237721, 1:4000) visualized with iFluor 430 Tyramide (AAT Bioquest, 45096, 1:400); CD8 (ZSGB-BIO, ZA-0508, ready-to-use) visualized with iFluor 488 Tyramide (AAT Bioquest, 11060, 1:30); and GZMB (Abcam, ab255598, 1:8000) visualized with CY3 Tyramide (AAT Bioquest, 11065, 1:600). The corresponding secondary antibodies were IgG H&L (HRP) (Abcam, ab205718, 1:4000). Nuclei were stained with DAPI (Solarbio, C0060). After staining, the slides were digitized using the Pannoramic SCAN II (3D HISTECH). Regions of interest (ROIs) were selected in CaseViewer 2.4, and images of these areas were imported into Visiopharm for quantitative image analysis. The tumor core, tumor margin, and stromal regions were selected for analysis due to their distinct impacts on tumor cells [55]. The regions were delineated based on specific principles: the tumor region predominantly contains tumor cells, the stromal region is primarily composed of stromal cells, and the margin region is defined as a band ~250 µm wide around the tumor-stroma interface, containing a mix of tumor and stromal elements. For each sample, at least two areas within each of these three regions were selected to calculate the mean expression levels. H&E staining was performed on consecutive sections that had undergone IF staining to accurately assess these regions. The number of various cell populations was quantified as the number of stained cells per square millimeter.

### Immunohistochemistry (IHC) staining and evaluation

FFPE tumor tissue blocks were serially sectioned into 5 μm slices. The processes of deparaffinization, rehydration, antigen retrieval, and endogenous peroxidase blocking were carried out under the same conditions as those used in the IF experiments of tissues. Following these preparations, the sections were blocked and subsequently incubated with the anti-HSD17B12 antibody (Abcam, ab236990, 1:900) overnight at 4 °C, followed by incubation with the secondary antibody (Abcam, ab205718, 1:2000) according to standard procedures. Antibody binding was visualized using the Ultra DAB kit, and nuclei were counterstained with hematoxylin for 1 min. Images were acquired using the NanoZoomer S360 digital slide scanner. For analysis, QuPath software (RRID: SCR_018257) was employed. Initially, three regions within the tumor area of each sample were selected for detailed examination, with H&E staining performed on consecutive sections that had undergone IHC staining to precisely assess the tumor regions. Employing QuPath’s random forest model, the percentage of positive cells and the H-score for each selected region were calculated, and the average values across the three regions were determined. The H-score, a semi-quantitative method for assessing staining intensity and distribution in IHC [56], categorizes cells into four staining intensity levels: 0 (no staining), 1+ (weak), 2+ (moderate), and 3+ (strong). The percentage of cells in each category (P0, P1, P2, and P3) was determined, and the H-score was calculated using the formula: H-score = (P1 × 1) + (P2 × 2) + (P3 × 3). This formula yields a score ranging from 0 to 300, where a score of 0 indicates no staining and a score of 300 indicates that 100% of the cells exhibit strong staining.

### Statistical analysis

Statistical analysis was performed using GraphPad Prism 7.0 (GraphPad Software Inc, San Diego, California, USA). Quantitative results are reported as the mean ± SD or mean ± SEM as indicated in the figure legends. All tests were Two-tailed, and *P* < 0.05 was considered statistically significant.

## Supporting information

S1 Fig HSD17B12 is an interaction partner of PD-L1 in live cells. (A) Flow chart for identifying PD-L1 binding candidates using the PUP-IT system. (B) Expression of the PD-L1-Flag-PafA in HEK293T cells. Three biological replicates. (C) List of potential PD-L1-interacting candidates in cells. (D and E) HSD17B12 co-immunoprecipitates with PD-L1 in MDA-MB-231 cells (D) and RKO cells (E). Experiments in D and E were repeated three times independently with similar results. (F) RKO monoclonal cells with in situ Flag knock-in at the endogenous *HSD17B12* locus. The successful knock-in of the Flag tag resulted in a shift of HSD17B12, and the tagged protein was detected using an anti-Flag antibody. (G) Representative images showing PD-L1 partially colocalizes with HSD17B12 in MDA-MB-231 cells (scale bars: 5 μm). (H) Prediction of transmembrane domains in HSD17B12. Numerical data of (G) can be found in S2 Data, sheet “S1 Fig”.
(TIF)

S2 Fig HSD17B12 negatively regulates PD-L1 stability. (A and B) HSD17B12 knockdown in cancer cells. (A) RT-qPCR results show that HSD17B12 KD reduces HSD17B12 mRNA levels in A375 and HCT 116 cells. Values are mean ± SD from three independent experiments. The statistical differences were determined by a two-tailed Student *t* test. (B) Protein level of HSD17B12 decreases in the HSD17B12 KD HCT 116 and SK-MEL-28 cells. The experiment was performed three times. (B-D) HSD17B12 KD elevates PD-L1 levels in cancer cells. Experiments in B–D were repeated three times independently with similar results. (E–G) HSD17B12 influences PD-L1 expression in the presence of IFNγ. Cells were treated with 10 ng/mL IFN-γ for 48 hours. Experiments in E–G were repeated three times independently with similar results. (H and I) HSD17B12 deficiency results in increased plasma membrane-located PD-L1 in A375 cells. A375 cells and HSD17B12 KD A375 cells were collected for flow cytometry (H) and immunoblotting (I). Experiments in H and I were repeated three times independently with similar results. (J) HSD17B12 deficiency does not affect MHC-I expression in A375 cells. Cells were treated with 10 ng/mL IFNγ for 48 hours before collection. Three biological replicates. Numerical data of (A) and (H) can be found in S2 Data, sheet “S2 Fig”.
(TIF)

S3 Fig HSD17B12 promotes PD-L1 degradation via a lysosomal-dependent mechanism. (A) HSD17B12 regulates PD-L1 independently of the proteasome pathway. HCT 116 cells and HSD17B12 KD HCT 116 cells were treated with 30 μM MG132 for 6 hours before immunoblotting analysis. The experiment was performed three times. (B) Ubiquitination level of PD-L1 was not influenced by HSD17B12 KD in RKO cells. Three biological replicates. (C) HSD17B12 regulates PD-L1 in WM266−4 cells dependent on the lysosome. WM266−4 cells and HSD17B12 KD WM266−4 cells were incubated with CQ and 10 ng/mL IFNγ for 24 hours. The experiment was performed three times. (D and E) Immunofluorescence results reveal that HSD17B12 KD does not impact the colocalization of PD-L1 and RAB7B in A375 cells. (D) Representative images are displayed (scale bars: 5 μm). (E) The statistical analysis of the PD-L1-RAB7B colocalization factor (Pearson’s R value). The statistics were presented as mean ± SD and compared using a two-tailed Student *t* test. (F and G) HSD17B12 KD has no effect on the colocalization of PD-L1 and RAB11 in A375 cells. (F) Representative images are shown (scale bars: 5 μm). (G) The statistical analysis of the PD-L1-RAB11 colocalization factor (Pearson’s R value). Statistics were plotted as mean ± SD and compared using a two-tailed Student *t* test. (H) Monoclone isolated from RKO cells transduced with an sgRNA-targeting ATG7 was analyzed by immunoblot. (I) Lysotracker Red staining indicated that HSD17B12 does not affect acidic lysosomes morphology in living cells (scale bars: 5 μm). Numerical data of (E) and (G) can be found in S2 Data, sheet “S3 Fig”.
(TIF)

S4 Fig Characterization of HSD17B12-mediated PD-L1 degradation. (A) Interaction between PD-L1 and CMTM6 is unaffected by HSD17B12 KD. Immunoblots for input and IP were presented. Three biological replicates. (B) HSD17B12 KD does not affect the interaction between PD-L1 and HIP1R. The experiment was performed three times. (C) CMTM6 KO does not affect PD-L1 interaction with HSD17B12. Three biological replicates. (D and E) Immunofluorescence showing the PD-L1-HSD17B12 colocalization is not affected by CMTM6 KO in RKO cells. (D) Immunofluorescence staining was performed using the anti-PD-L1 and anti-CMTM6 antibodies. Scale bars: 5 μm. (E) The statistical outcome of the colocalization factor (Pearson’s R value). Statistics were shown as mean ± SD and compared using a two-tailed Student *t* test. (F and G) HSD17B12 regulates PD-L1 in RKO cells independently of CMTM6. Cells were harvested for immunoblotting analysis (F) and flow cytometry (G). Experiments in F and G were repeated three times independently with similar results. (H) A list of potential HSD17B12-dificiency-mediated differentiated PD-L1 interacting candidates. (I-K) HSD17B12 KD does not alter PD-L1 interaction with RAB13, COG2, and PPM1F. Cells co-expressing PD-L1-Strep II and Flag-RAB13 (I), Flag-COG2 (J), or Flag-PPM1F (K) were collected for the Flag-IP assay. Experiments in I–K were repeated three times independently with similar results. (L) Immunoblotting results demonstrate efficient VAC14 KD in RKO cells. The experiment was performed three times. (M) HRS significantly decreased in the HRS KD RKO cells. Three biological replicates. (N) Expression of VPS28 decreased in VPS28 KD RKO cells. Representative of three experiments. (O) HSD17B12 regulates PD-L1 stability in RKO cells dependent on VPS28. The experiment was performed three times. Numerical data of (E), (G), and (H) can be found in S2 Data, sheet “S4 Fig”.
(TIF)

S5 Fig Cys272 palmitoylation is not required for HSD17B12-mediated PD-L1 degradation. (A) Schematics of PD-L1 mutants used for mapping the HSD17B12-binding region. (B) The R265-C272 motif of PD-L1 plays an important role in its interaction with HSD17B12. Immunoblots for input and IP were shown. Three biological replicates. (C) Interaction between PD-L1_C272A_ mutant and HSD17B12 is significantly reduced. The experiment was performed three times. (D) Monoubiquitination level of PD-L1 is not affected by HSD17B12 KD in RKO cells. Cells expressing PD-L1-Flag were treated with 50 μM chloroquine (CQ) for 24 hours. The PD-L1-Flag proteins were immunoprecipitated using Flag beads and blotted with an anti-Ub antibody. The experiment was performed three times. (E) HSD17B12 KD has no effect on PD-L1 palmitoylation in RKO cells. Three biological replicates. (F and G) Cys272 palmitoylation is not required for HSD17B12-mediated PD-L1 regulation. The GAPDH level was detected as a loading control. (F) HSD17B12 KD influences the expression of the PD-L1_C272A_ mutant. Representative of three experiments. (G) HSD17B12 KD affects PD-L1 expression in RKO cells treated with 2-BP. Cells were incubated with 2-BP for 24 hours. The experiment was performed three times. (H) Stability of PD-L1-Ub in RKO cells is affected by HSD17B12 knockdown. Three biological replicates.
(TIF)

S6 Fig Mapping the PD-L1-interacting region in HSD17B12 and creating peptide stimulates PD-L1 degradation. (A) Schematics of the designed HSD17B12 truncations. (B–D) Mapping of the interaction between PD-L1 and HSD17B12 mutants. Immunoblots for input and IP were shown. Experiments in B–D were repeated three times independently with similar results. (E) Constructs of the HSD17B12 mimic peptides. (F and G) Characterize the potential of HSD17B12 mimic peptides to induce PD-L1 degradation. Experiments in F and G were repeated three times independently with similar results. (H) The CC1-NPGY lowered the stability of PD-L1 in RKO cells. Cells were treated with 100 μg/mL CHX for the indicated hours. Three biological replicates. (I) HSD17B12 KD significantly induces mPD-L1 expression in CT26 cells. The experiment was performed three times. (J) Immunoblotting results show that CC1-NPGY decreased exosomal PD-L1 level in CT26 cells. The experiment was performed three times. (K–M) CC1-NPGY does not affect CT26 tumor growth in BALB/c-Nude mice as evidenced by tumor images (K), statistical analysis of tumor volumes (L), and tumor weight (M) from different groups (*n* = 6 per group). Values indicate mean ± SEM in (L) and mean ± SD in (M), compared by a two-tailed Student *t* test. (N) CC1-NPGY fails to suppress tumor growth of mPD-L1 KO cancer cells in BALB/c mice, as shown by tumor images (*n* = 6 per group). Numerical data of (L) and (M) can be found in S2 Data, sheet “S6 Fig”.
(TIF)

S1 Data Numerical values of data in Figs 1–6. (XLSX)

S2 Data Numerical values of data in S1–S6 Figs. (XLSX)

S1 Raw Images Raw images in Figs 1–6 and S1–S6. (PDF)

S1 Table Classification based on HSD17B12 relative protein expression levels. List of HSD17B12 expression in tumor tissues and paired normal tissues.
(DOCX)

S2 Table Clinical information for CRC cohort. List of clinical characteristics for the colorectal cancer patients, including age, sex, overall survival, and tumor location.
(DOCX)

## Abbreviations

2-BP: 2-bromopalmitate
ABE: acyl-biotin exchange
Ala: alanine
CHX: cycloheximide
CRC: colorectal cancer
DAPI: 4′,6-diamidino-2-phenylindole
dMMR: deficient mismatch repair
DMEM: Dulbecco’s modified Eagle’s medium
ERAD: ER-associated degradation
ESCRT: endosomal sorting complexes required for transport
exoPD-L1: exosomal PD-L1
FBS: fetal bovine serum
FFPE: formalin-fixed, paraffin-embedded
GzmB^+^: granzyme B-positive^+^
HIP1R: huntington-interacting protein 1-related
IF: immunofluorescence
IHC: immunohistochemistry
MFI: mean fluorescence intensity
MHC-I: MHC class I
MVBs: multivesicular-bodies
NSCLC: nonsmall-cell lung carcinoma
OS: overall survival
PBMCs: peripheral blood mononuclear cells
PUP-IT: pupylation-based interaction tagging
RCC: renal cell carcinoma
ROIs: regions of interest
RT-qPCR: reverse transcription quantitative real-time PCR
ssGSEA: single-sample gene set enrichment analysis
TRG2: tumor regression grade 2
