# Supplementary figures and images for: The lipid-metabolic enzyme HSD17B12 drives lysosomal degradation of PD-L1 potentiating anti-tumor immunity in a mouse model
Source: PLoS Biol. 2026 Jan 27;24(1):e3003603. doi: 10.1371/journal.pbio.3003603 (PMC12843542; doi:10.1371/journal.pbio.3003603)

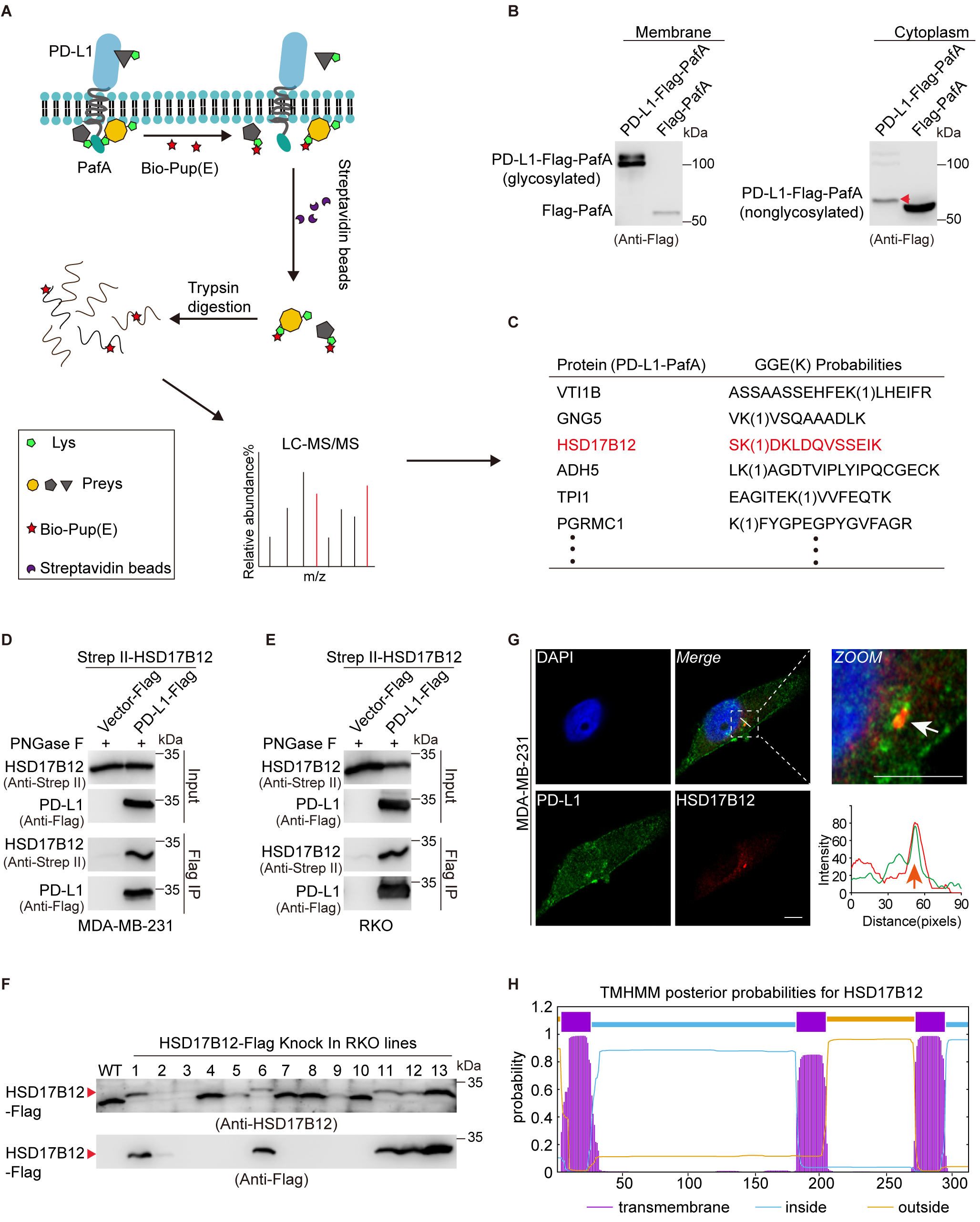

Supplement: S1 Fig — (A) Flow chart for identifying PD-L1 binding candidates using the PUP-IT system. (B) Expression of the PD-L1-Flag-PafA in HEK293T cells. Three biological replicates. (C) List of potential PD-L1-interacting candidates in cells. (D and E) HSD17B12 co-immunoprecipitates with PD-L1 in MDA-MB-231 cells (D) and RKO cells (E). Experiments in D and E were repeated three times independently with similar results. (F) RKO monoclonal cells with in situ Flag knock-in at the endogenous HSD17B12 locus. The successful knock-in of the Flag tag resulted in a shift of HSD17B12, and the tagged protein was detected using an anti-Flag antibody. (G) Representative images showing PD-L1 partially colocalizes with HSD17B12 in MDA-MB-231 cells (scale bars: 5 μm). (H) Prediction of transmembrane domains in HSD17B12. Numerical data of (G) can be found in S2 Data, sheet “S1 Fig”. (TIF) [file pbio.3003603.s001.tif]

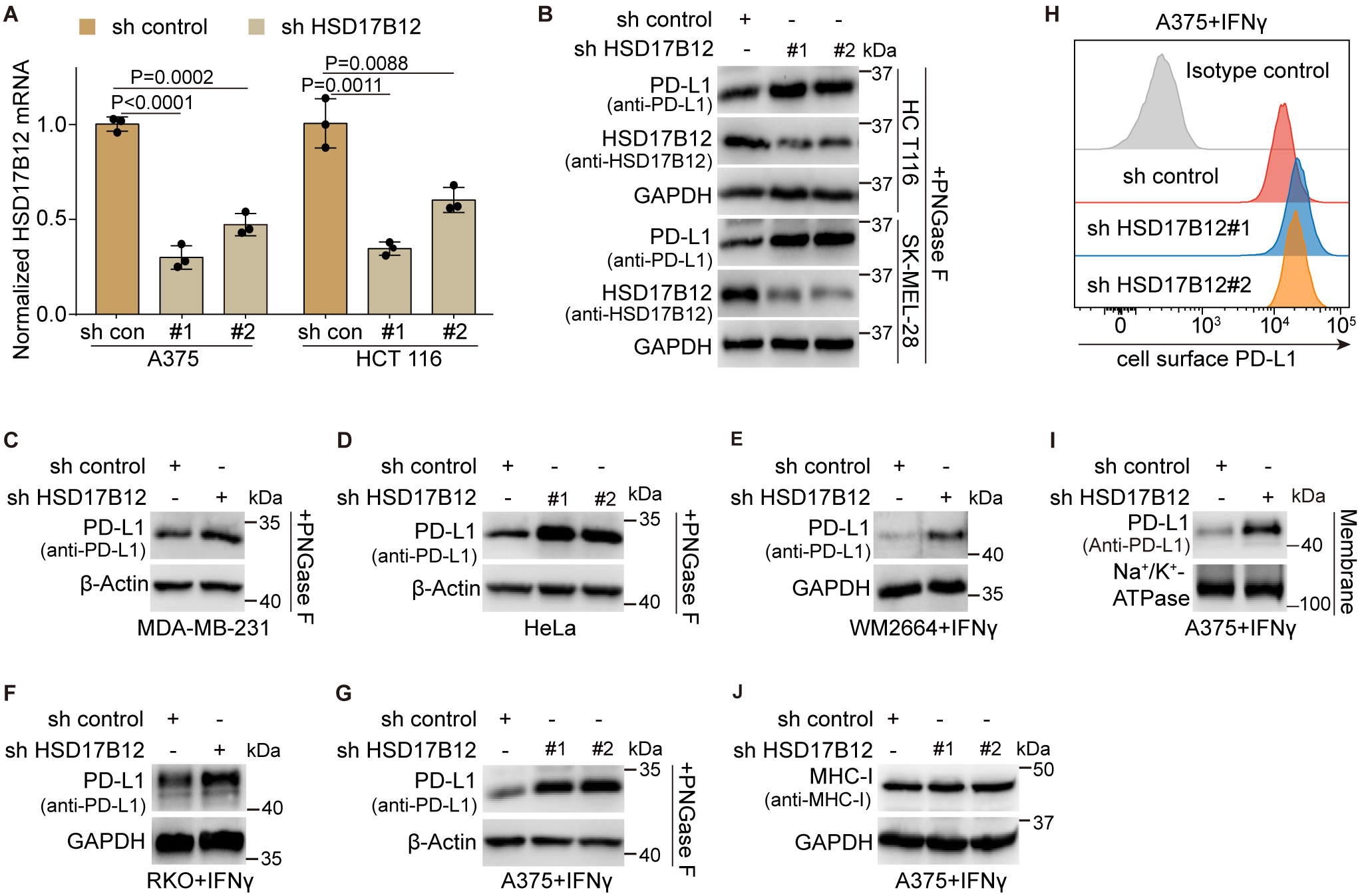

Supplement: S2 Fig — (A and B) HSD17B12 knockdown in cancer cells. (A) RT-qPCR results show that HSD17B12 KD reduces HSD17B12 mRNA levels in A375 and HCT 116 cells. Values are mean ± SD from three independent experiments. The statistical differences were determined by a two-tailed Student t test. (B) Protein level of HSD17B12 decreases in the HSD17B12 KD HCT 116 and SK-MEL-28 cells. The experiment was performed three times. (B-D) HSD17B12 KD elevates PD-L1 levels in cancer cells. Experiments in B–D were repeated three times independently with similar results. (E–G) HSD17B12 influences PD-L1 expression in the presence of IFNγ. Cells were treated with 10 ng/mL IFN-γ for 48 hours. Experiments in E–G were repeated three times independently with similar results. (H and I) HSD17B12 deficiency results in increased plasma membrane-located PD-L1 in A375 cells. A375 cells and HSD17B12 KD A375 cells were collected for flow cytometry (H) and immunoblotting (I). Experiments in H and I were repeated three times independently with similar results. (J) HSD17B12 deficiency does not affect MHC-I expression in A375 cells. Cells were treated with 10 ng/mL IFNγ for 48 hours before collection. Three biological replicates. Numerical data of (A) and (H) can be found in S2 Data, sheet “S2 Fig”. (TIF) [file pbio.3003603.s002.tif]

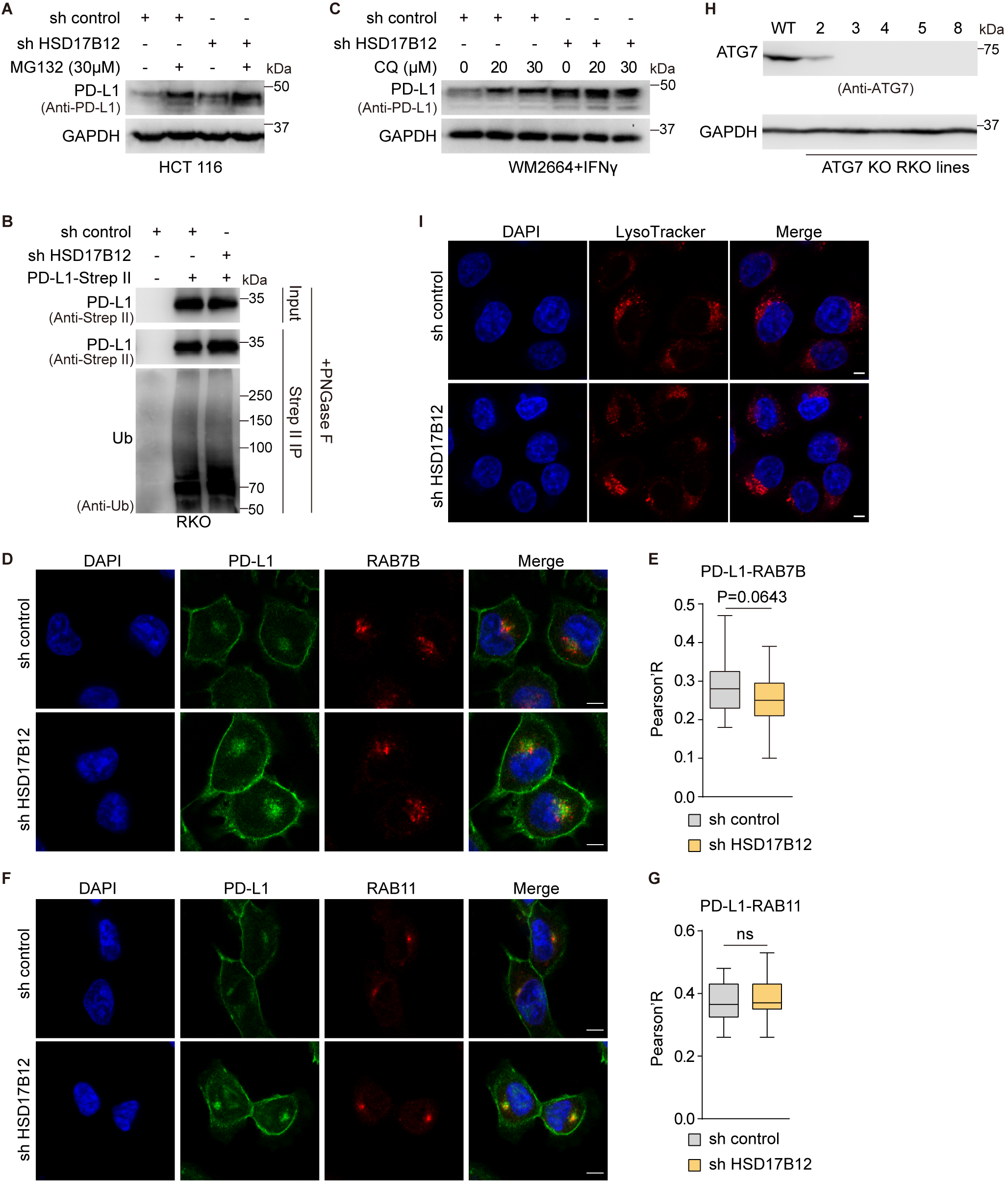

Supplement: S3 Fig — (A) HSD17B12 regulates PD-L1 independently of the proteasome pathway. HCT 116 cells and HSD17B12 KD HCT 116 cells were treated with 30 μM MG132 for 6 hours before immunoblotting analysis. The experiment was performed three times. (B) Ubiquitination level of PD-L1 was not influenced by HSD17B12 KD in RKO cells. Three biological replicates. (C) HSD17B12 regulates PD-L1 in WM266−4 cells dependent on the lysosome. WM266−4 cells and HSD17B12 KD WM266−4 cells were incubated with CQ and 10 ng/mL IFNγ for 24 hours. The experiment was performed three times. (D and E) Immunofluorescence results reveal that HSD17B12 KD does not impact the colocalization of PD-L1 and RAB7B in A375 cells. (D) Representative images are displayed (scale bars: 5 μm). (E) The statistical analysis of the PD-L1-RAB7B colocalization factor (Pearson’s R value). The statistics were presented as mean ± SD and compared using a two-tailed Student t test. (F and G) HSD17B12 KD has no effect on the colocalization of PD-L1 and RAB11 in A375 cells. (F) Representative images are shown (scale bars: 5 μm). (G) The statistical analysis of the PD-L1-RAB11 colocalization factor (Pearson’s R value). Statistics were plotted as mean ± SD and compared using a two-tailed Student t test. (H) Monoclone isolated from RKO cells transduced with an sgRNA-targeting ATG7 was analyzed by immunoblot. (I) Lysotracker Red staining indicated that HSD17B12 does not affect acidic lysosomes morphology in living cells (scale bars: 5 μm). Numerical data of (E) and (G) can be found in S2 Data, sheet “S3 Fig”. (TIF) [file pbio.3003603.s003.tif]

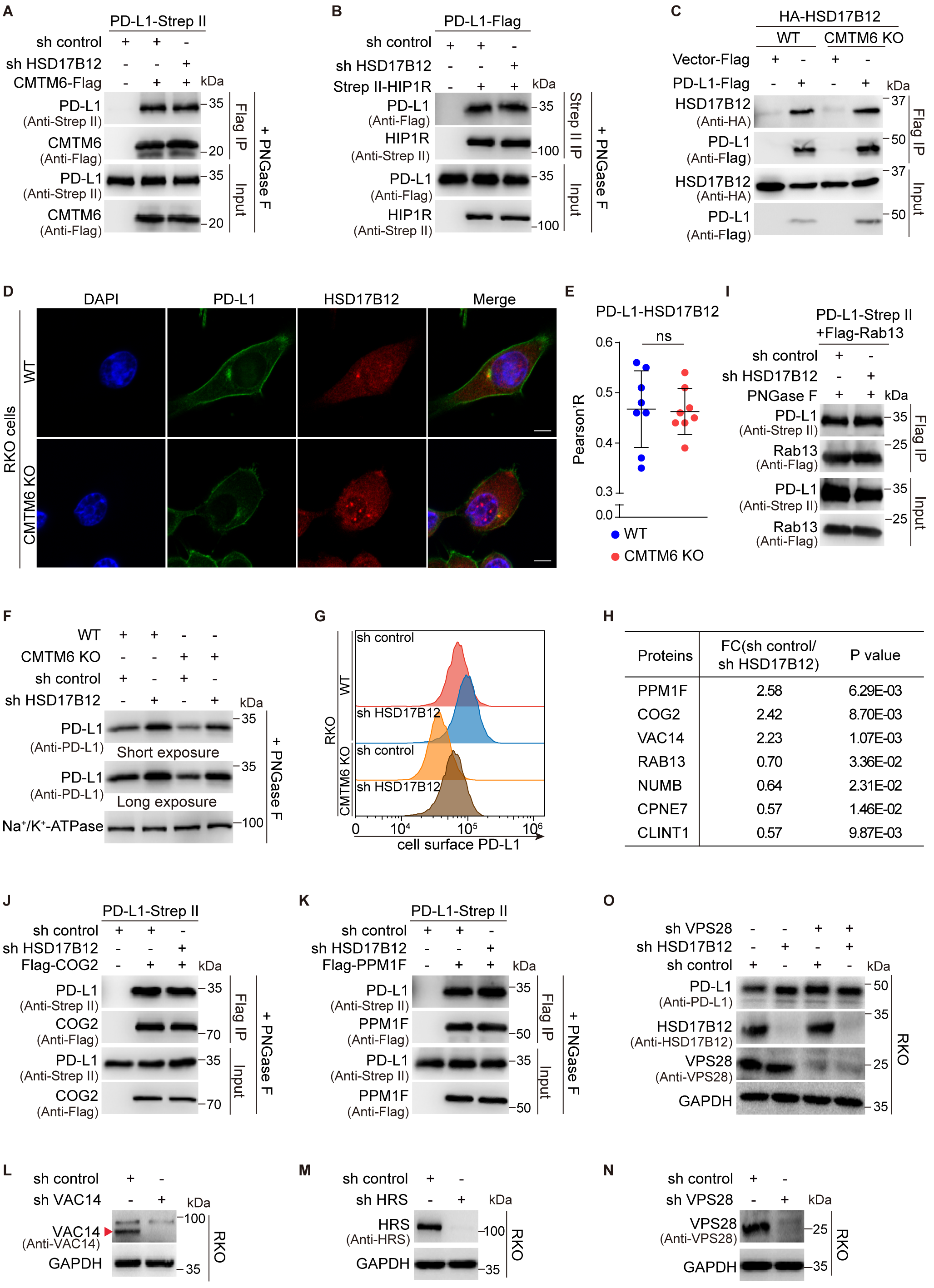

Supplement: S4 Fig — (A) Interaction between PD-L1 and CMTM6 is unaffected by HSD17B12 KD. Immunoblots for input and IP were presented. Three biological replicates. (B) HSD17B12 KD does not affect the interaction between PD-L1 and HIP1R. The experiment was performed three times. (C) CMTM6 KO does not affect PD-L1 interaction with HSD17B12. Three biological replicates. (D and E) Immunofluorescence showing the PD-L1-HSD17B12 colocalization is not affected by CMTM6 KO in RKO cells. (D) Immunofluorescence staining was performed using the anti-PD-L1 and anti-CMTM6 antibodies. Scale bars: 5 μm. (E) The statistical outcome of the colocalization factor (Pearson’s R value). Statistics were shown as mean ± SD and compared using a two-tailed Student t test. (F and G) HSD17B12 regulates PD-L1 in RKO cells independently of CMTM6. Cells were harvested for immunoblotting analysis (F) and flow cytometry (G). Experiments in F and G were repeated three times independently with similar results. (H) A list of potential HSD17B12-dificiency-mediated differentiated PD-L1 interacting candidates. (I-K) HSD17B12 KD does not alter PD-L1 interaction with RAB13, COG2, and PPM1F. Cells co-expressing PD-L1-Strep II and Flag-RAB13 (I), Flag-COG2 (J), or Flag-PPM1F (K) were collected for the Flag-IP assay. Experiments in I–K were repeated three times independently with similar results. (L) Immunoblotting results demonstrate efficient VAC14 KD in RKO cells. The experiment was performed three times. (M) HRS significantly decreased in the HRS KD RKO cells. Three biological replicates. (N) Expression of VPS28 decreased in VPS28 KD RKO cells. Representative of three experiments. (O) HSD17B12 regulates PD-L1 stability in RKO cells dependent on VPS28. The experiment was performed three times. Numerical data of (E), (G), and (H) can be found in S2 Data, sheet “S4 Fig”. (TIF) [file pbio.3003603.s004.tif]

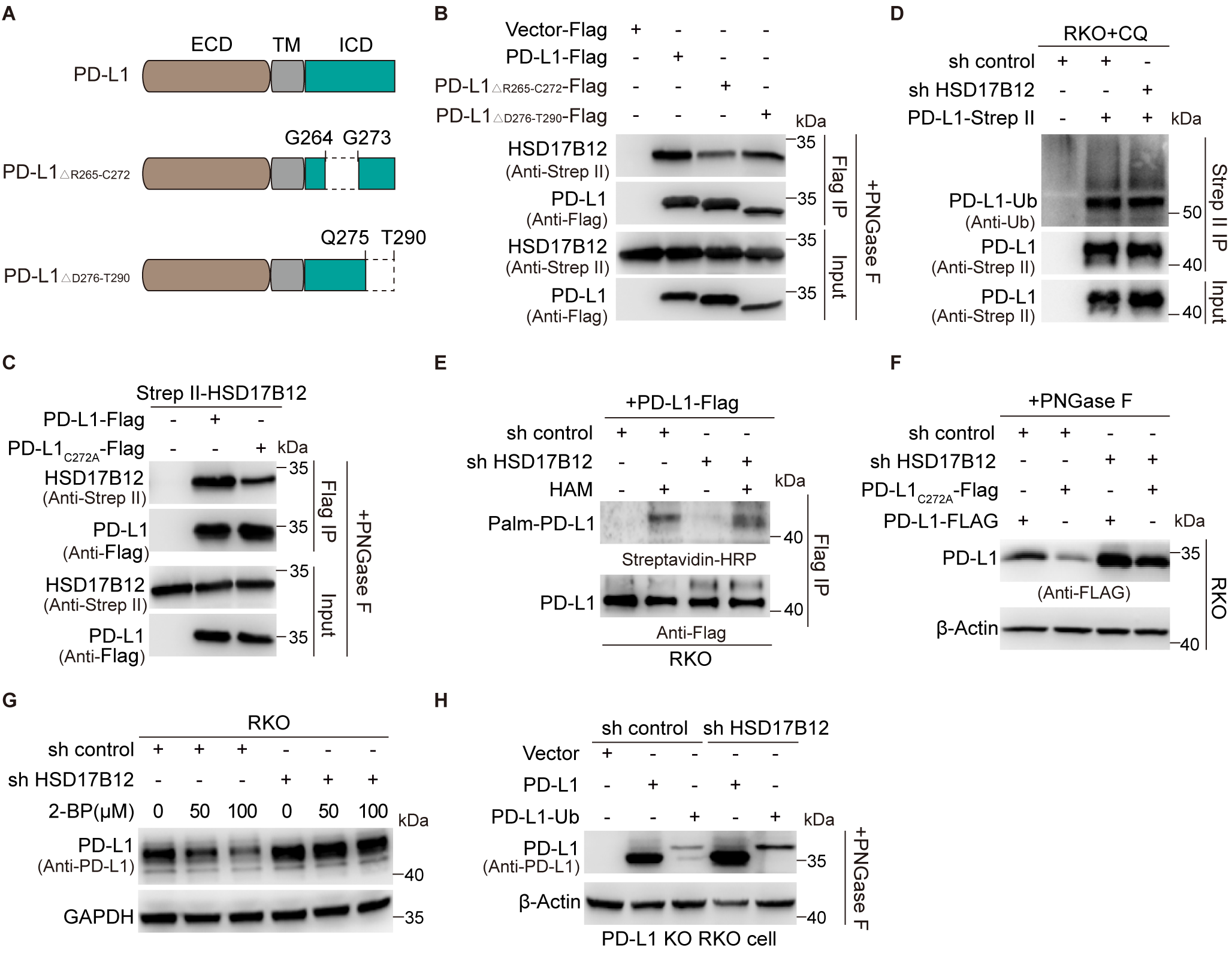

Supplement: S5 Fig — (A) Schematics of PD-L1 mutants used for mapping the HSD17B12-binding region. (B) The R265-C272 motif of PD-L1 plays an important role in its interaction with HSD17B12. Immunoblots for input and IP were shown. Three biological replicates. (C) Interaction between PD-L1C272A mutant and HSD17B12 is significantly reduced. The experiment was performed three times. (D) Monoubiquitination level of PD-L1 is not affected by HSD17B12 KD in RKO cells. Cells expressing PD-L1-Flag were treated with 50 μM chloroquine (CQ) for 24 hours. The PD-L1-Flag proteins were immunoprecipitated using Flag beads and blotted with an anti-Ub antibody. The experiment was performed three times. (E) HSD17B12 KD has no effect on PD-L1 palmitoylation in RKO cells. Three biological replicates. (F and G) Cys272 palmitoylation is not required for HSD17B12-mediated PD-L1 regulation. The GAPDH level was detected as a loading control. (F) HSD17B12 KD influences the expression of the PD-L1C272A mutant. Representative of three experiments. (G) HSD17B12 KD affects PD-L1 expression in RKO cells treated with 2-BP. Cells were incubated with 2-BP for 24 hours. The experiment was performed three times. (H) Stability of PD-L1-Ub in RKO cells is affected by HSD17B12 knockdown. Three biological replicates. (TIF) [file pbio.3003603.s005.tif]

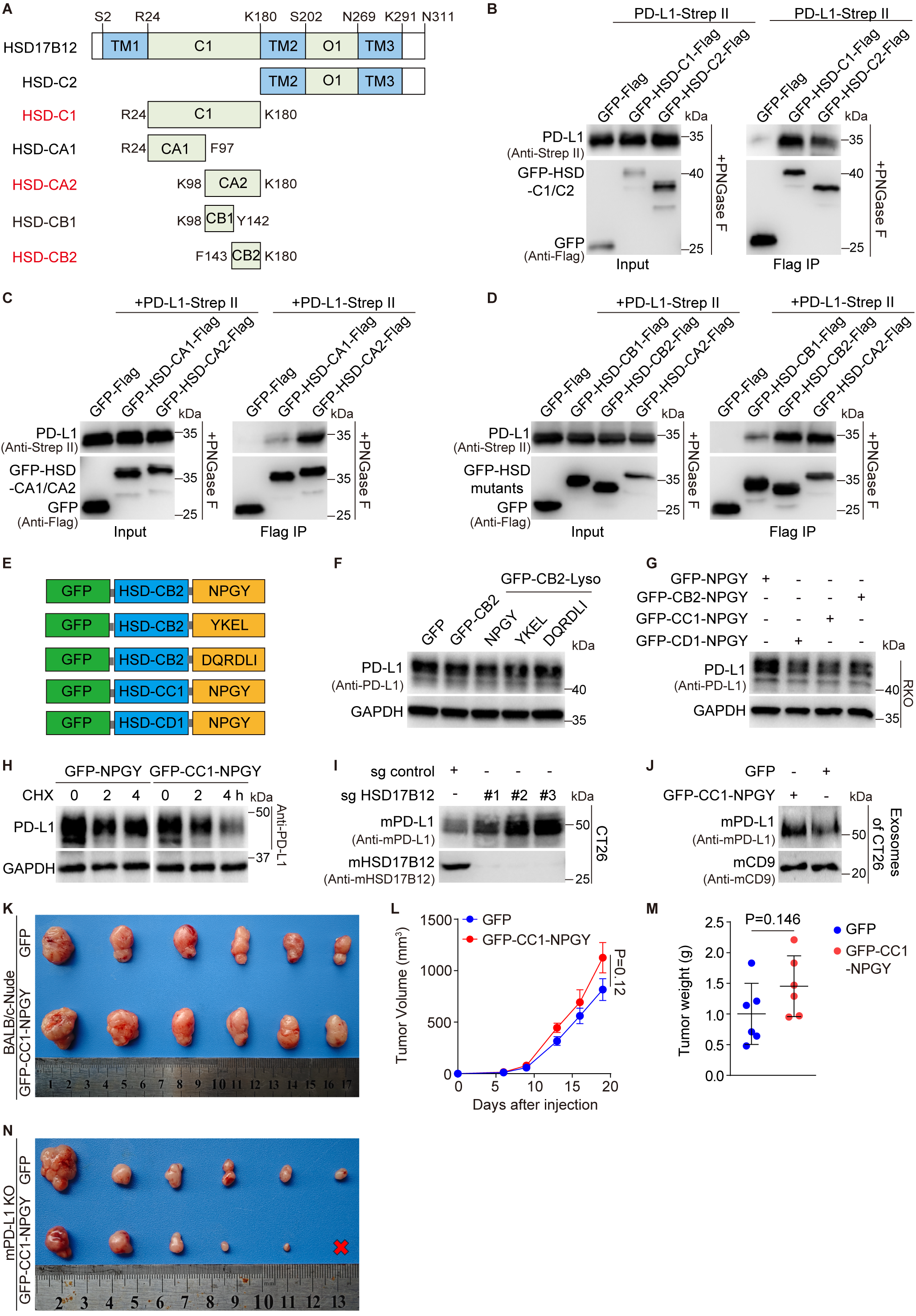

Supplement: S6 Fig — (A) Schematics of the designed HSD17B12 truncations. (B–D) Mapping of the interaction between PD-L1 and HSD17B12 mutants. Immunoblots for input and IP were shown. Experiments in B–D were repeated three times independently with similar results. (E) Constructs of the HSD17B12 mimic peptides. (F and G) Characterize the potential of HSD17B12 mimic peptides to induce PD-L1 degradation. Experiments in F and G were repeated three times independently with similar results. (H) The CC1-NPGY lowered the stability of PD-L1 in RKO cells. Cells were treated with 100 μg/mL CHX for the indicated hours. Three biological replicates. (I) HSD17B12 KD significantly induces mPD-L1 expression in CT26 cells. The experiment was performed three times. (J) Immunoblotting results show that CC1-NPGY decreased exosomal PD-L1 level in CT26 cells. The experiment was performed three times. (K–M) CC1-NPGY does not affect CT26 tumor growth in BALB/c-Nude mice as evidenced by tumor images (K), statistical analysis of tumor volumes (L), and tumor weight (M) from different groups (n = 6 per group). Values indicate mean ± SEM in (L) and mean ± SD in (M), compared by a two-tailed Student t test. (N) CC1-NPGY fails to suppress tumor growth of mPD-L1 KO cancer cells in BALB/c mice, as shown by tumor images (n = 6 per group). Numerical data of (L) and (M) can be found in S2 Data, sheet “S6 Fig”. (TIF) [file pbio.3003603.s006.tif]
