# Supplementary material for: The lipid-metabolic enzyme HSD17B12 drives lysosomal degradation of PD-L1 potentiating anti-tumor immunity in a mouse model
Source: PLoS Biol. 2026 Jan 27;24(1):e3003603. doi: 10.1371/journal.pbio.3003603 (PMC12843542; doi:10.1371/journal.pbio.3003603)

Figure 1G

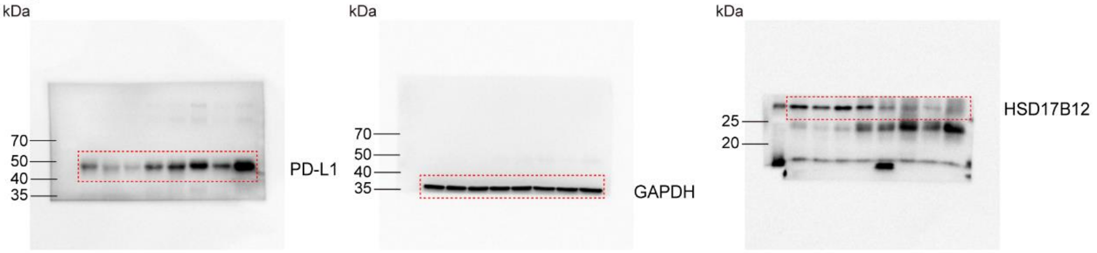

Figure 2A

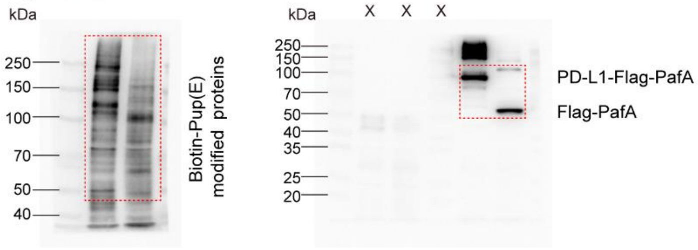

Figure 2C

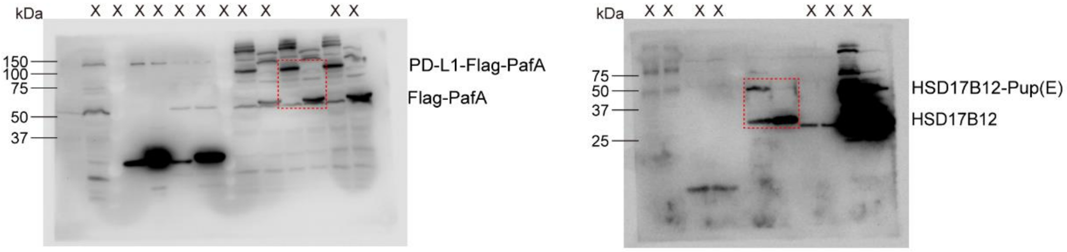

Figure 2D

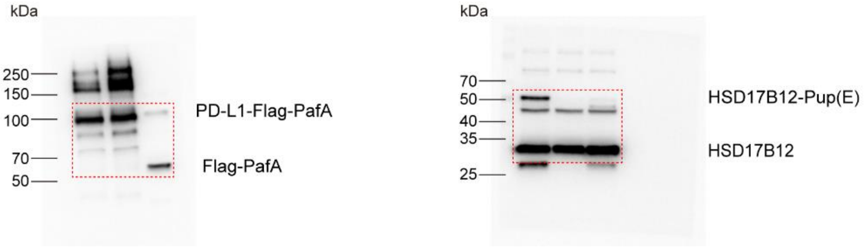

Figure 2E

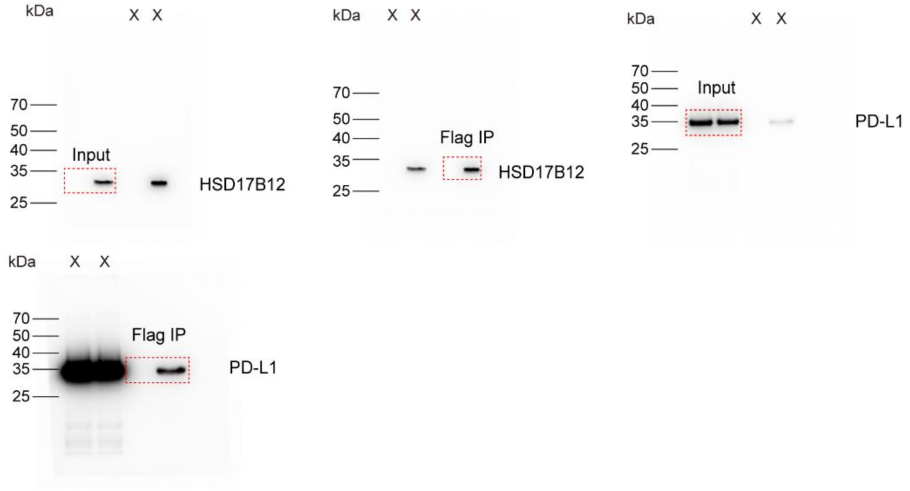

Figure 2F

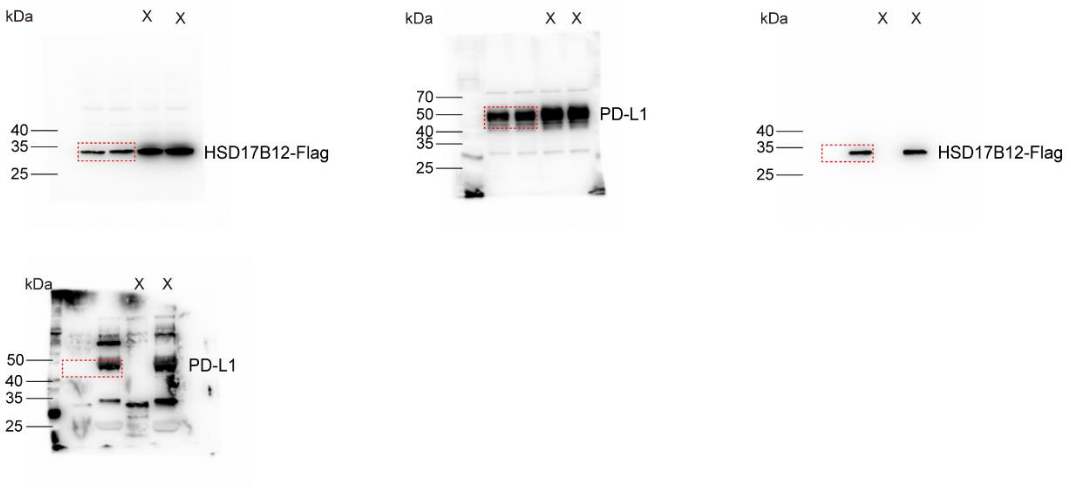

Figure 3A

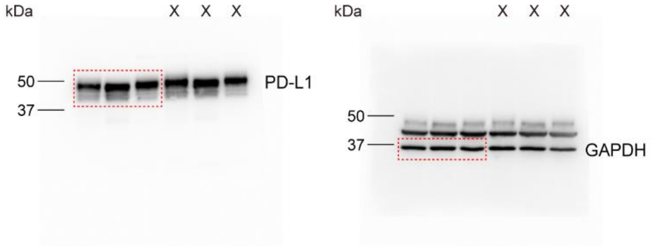

Figure 3B

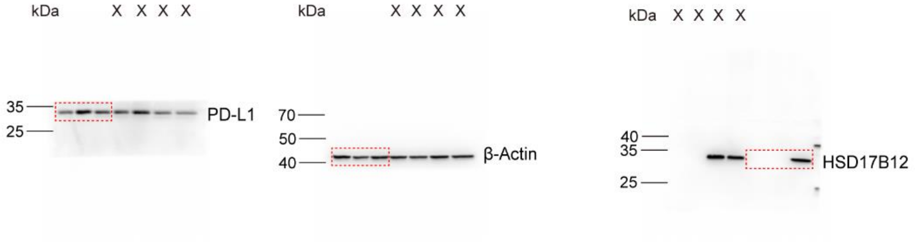

Figure 3C

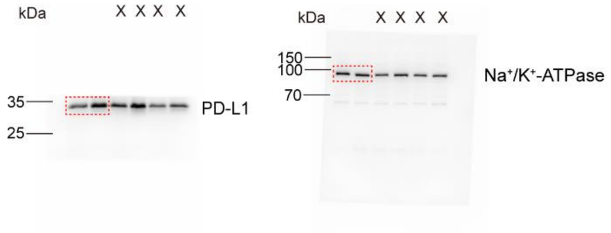

Figure 3F

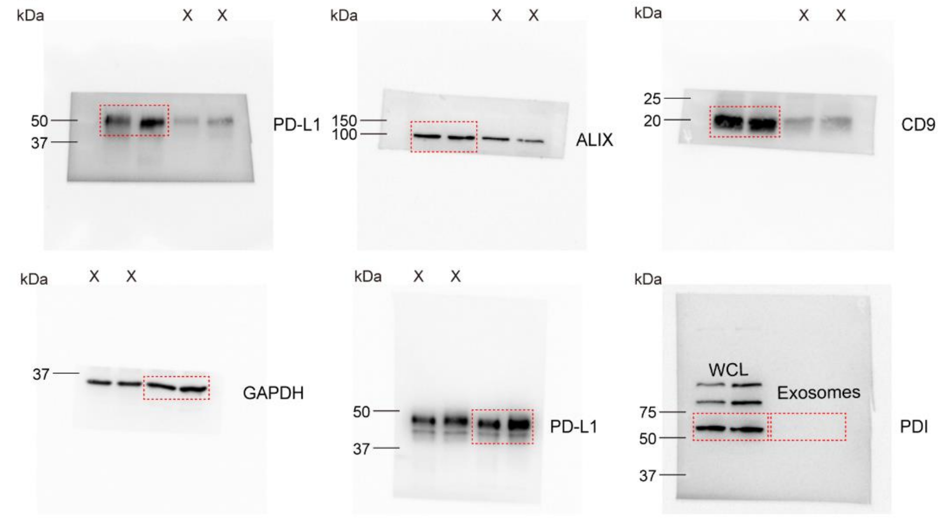

Figure 3G

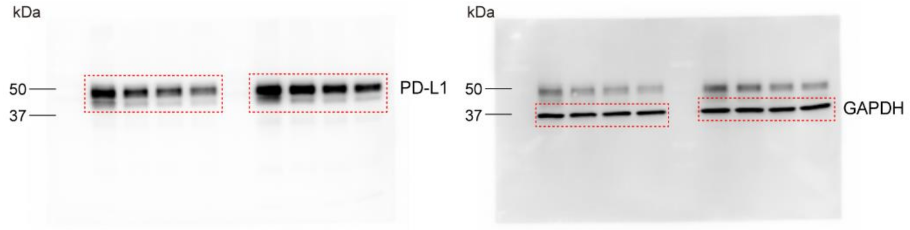

Figure 3H

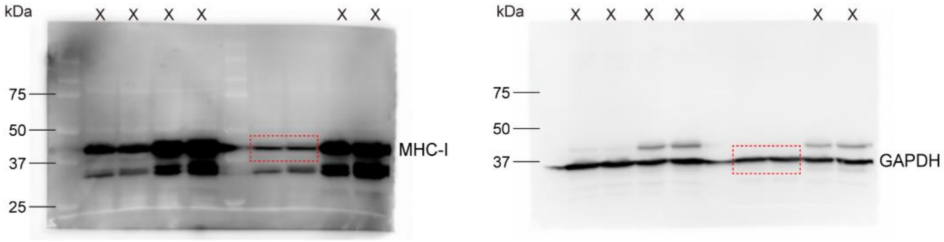

Figure 3I

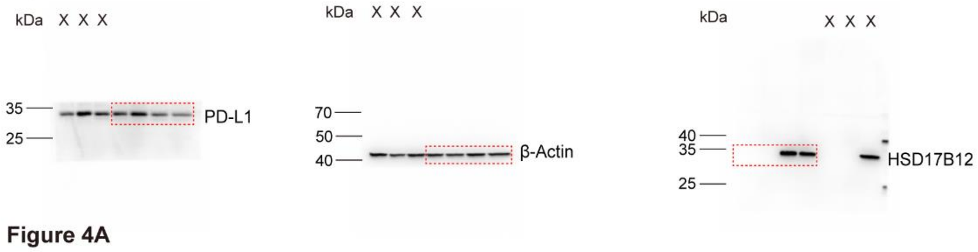

Figure 4A

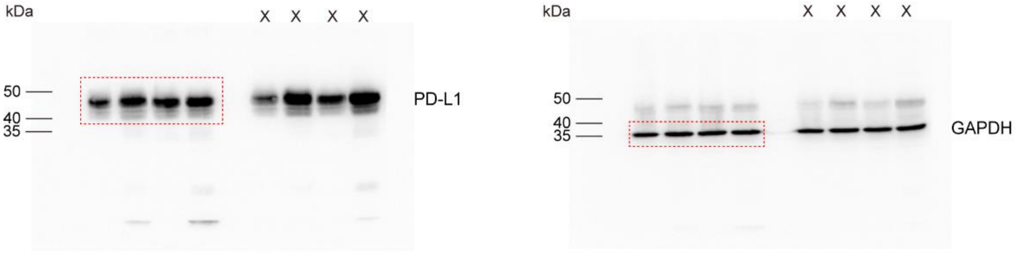

Figure 4E

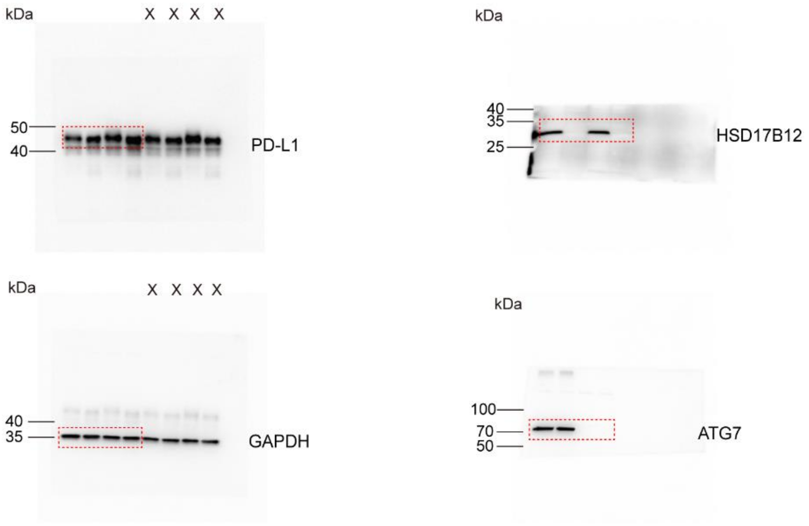

Figure 4G

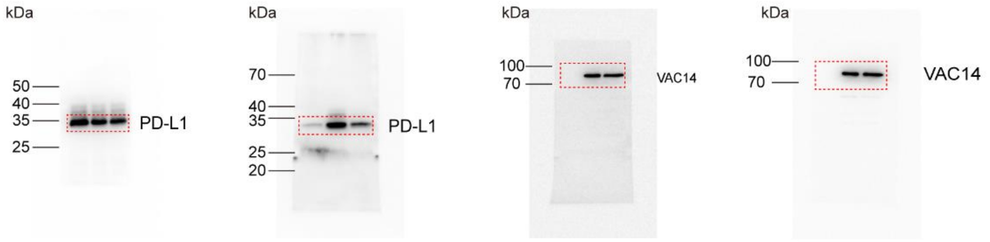

Figure 4H

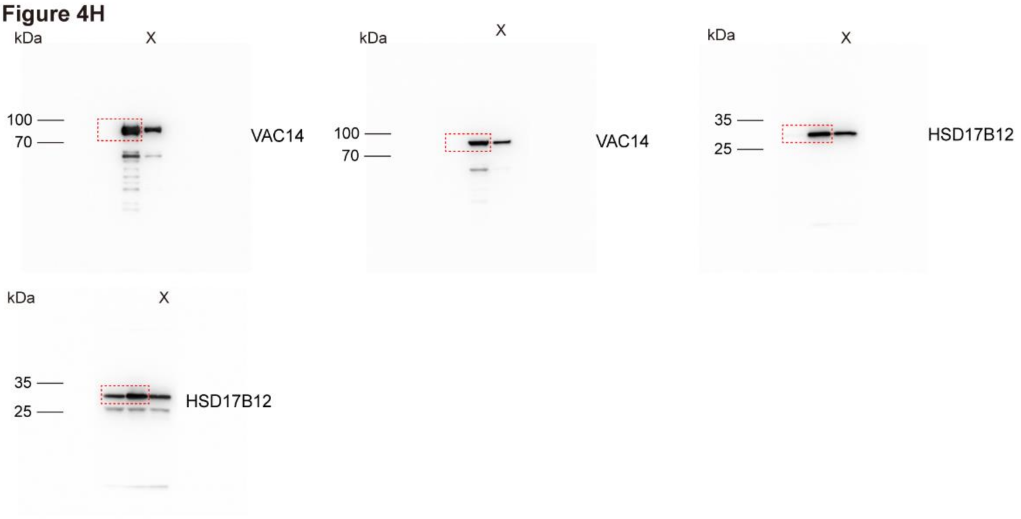

Figure 4I

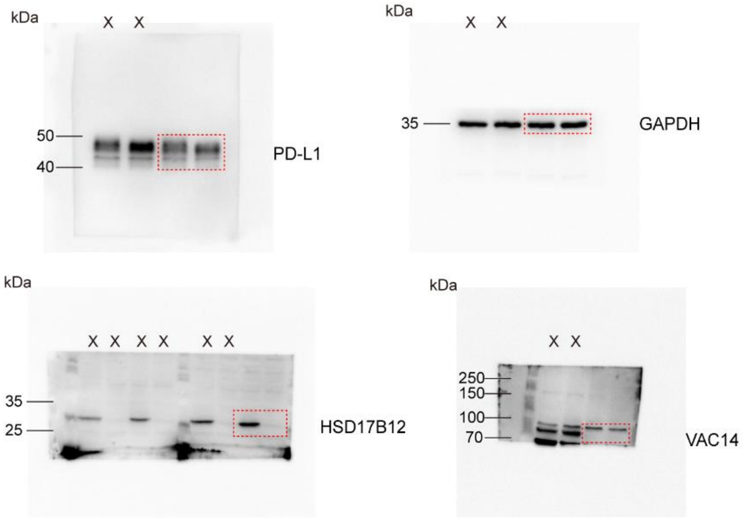

Figure 4K

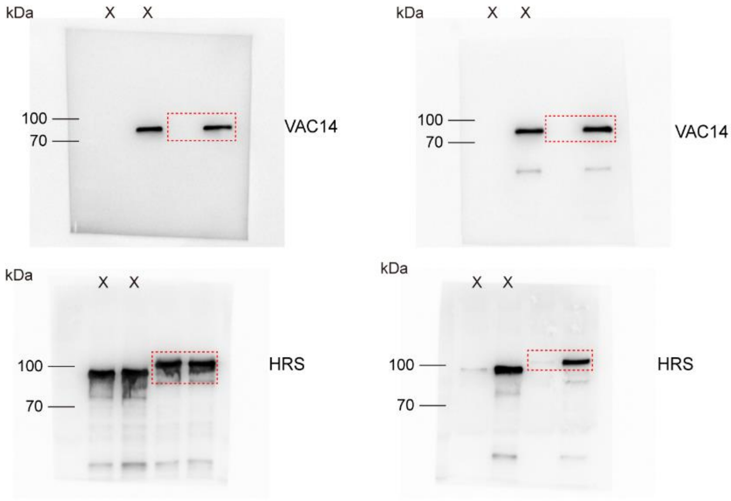

Figure 4L

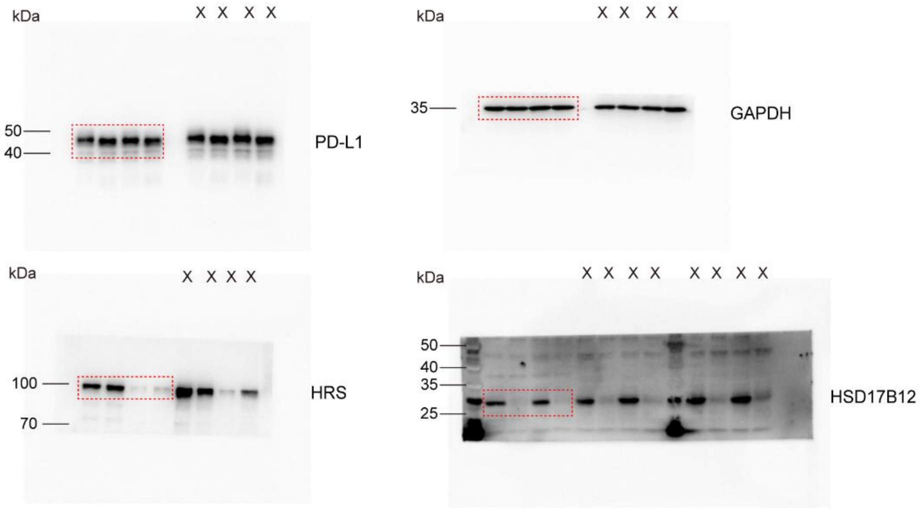

Figure 6B

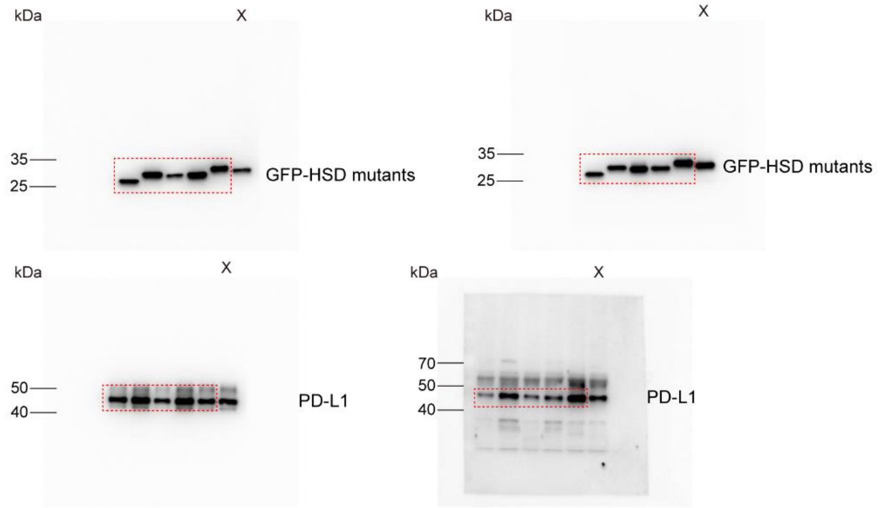

Figure 6D

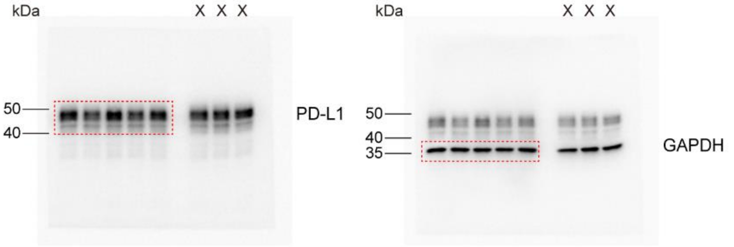

Figure 6G

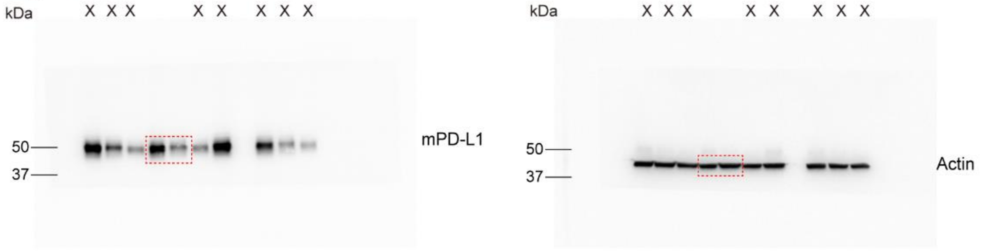

Figure 6L

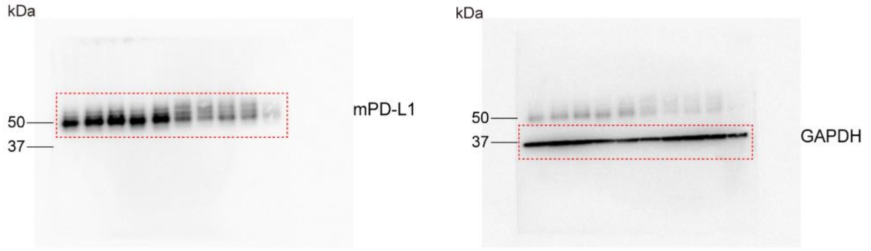

Figure 6M

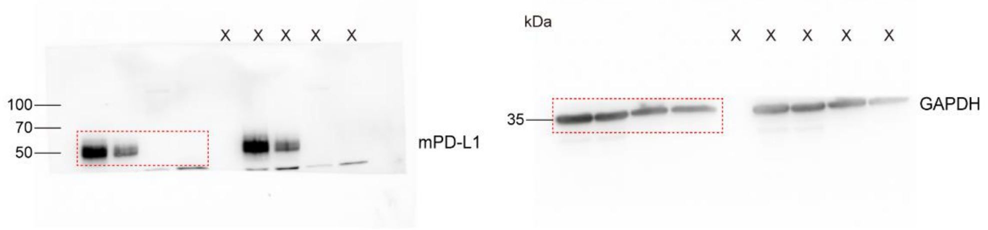

sFigure 1B

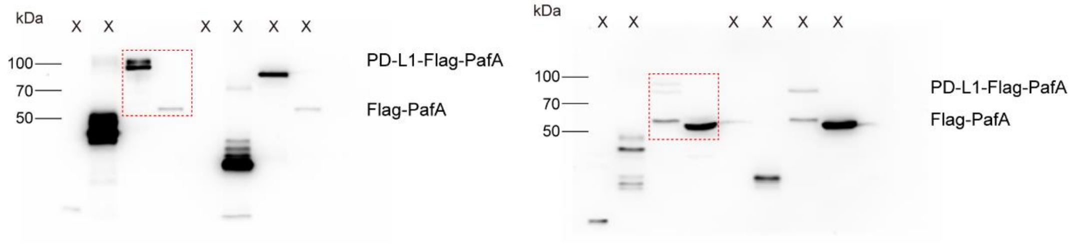

sFigure 1D

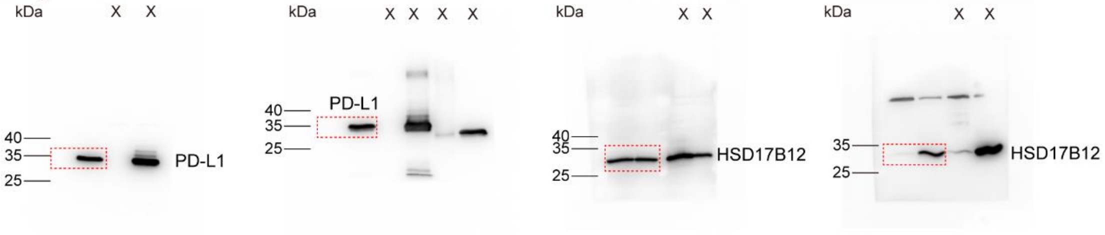

sFigure 1E

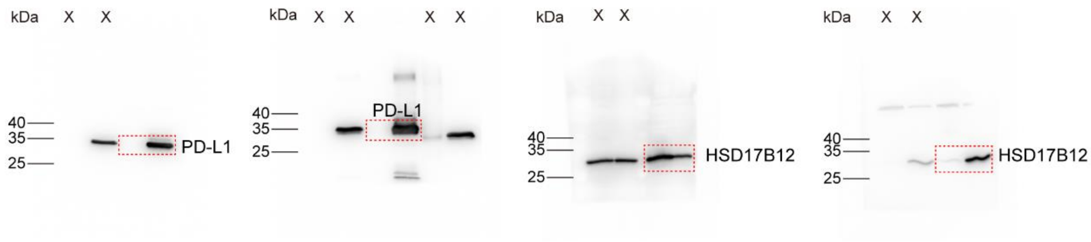

sFigure 1F

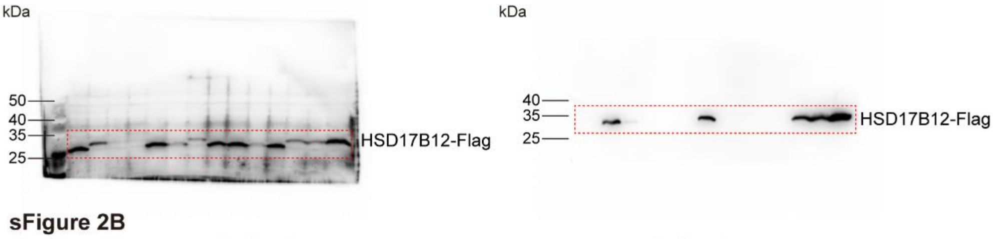

sFigure 2B

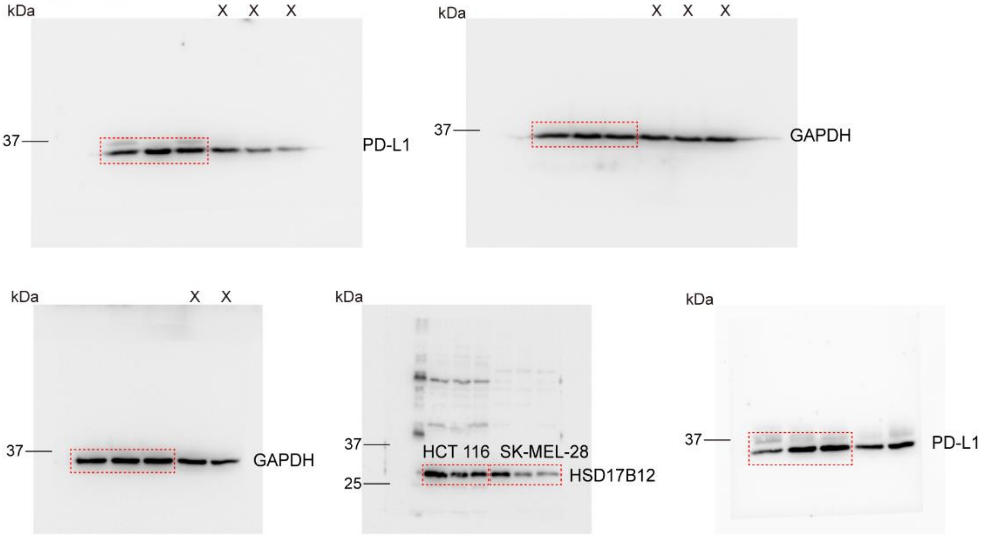

sFigure 2C

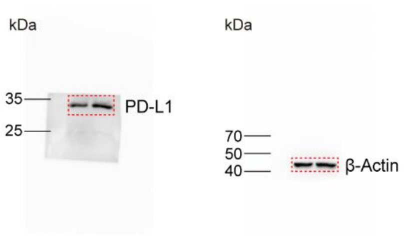

sFigure 2D

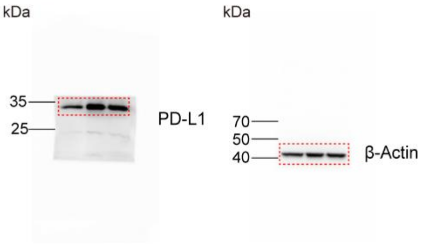

sFigure 2E

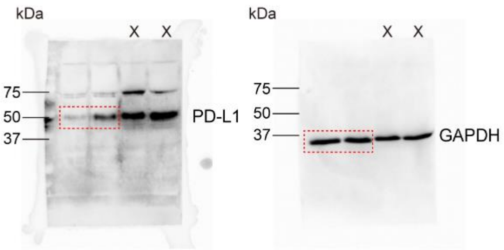

sFigure 2F

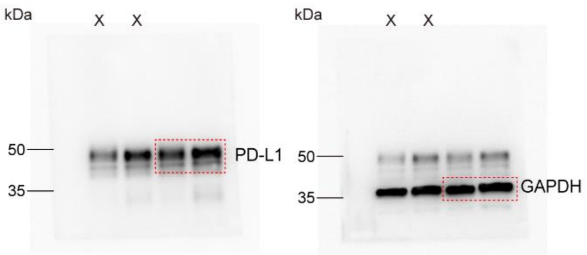

sFigure 2G

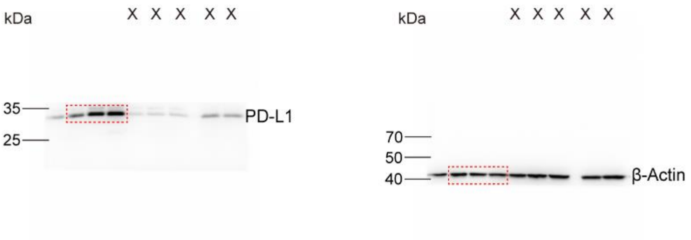

sFigure 2I

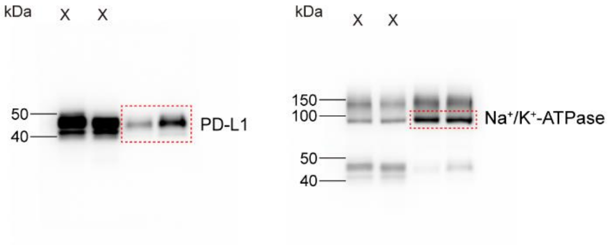

sFigure 2J

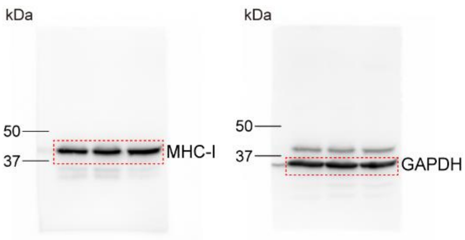

sFigure 3A

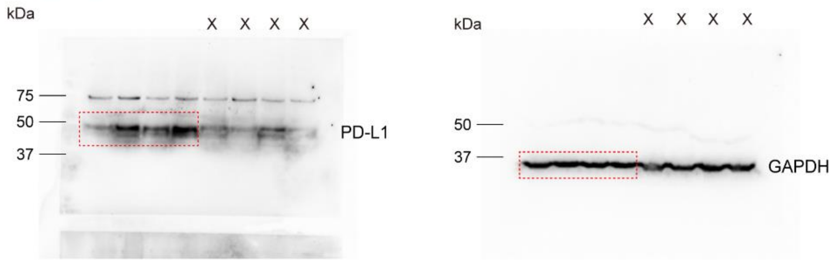

sFigure 3B

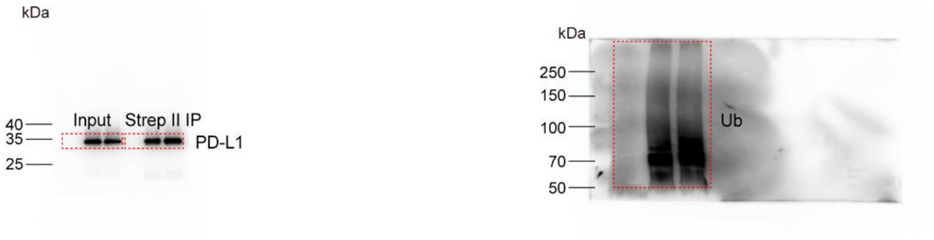

sFigure 3C

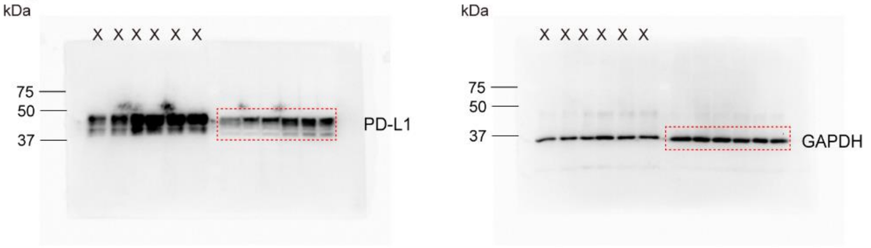

sFigure 3H

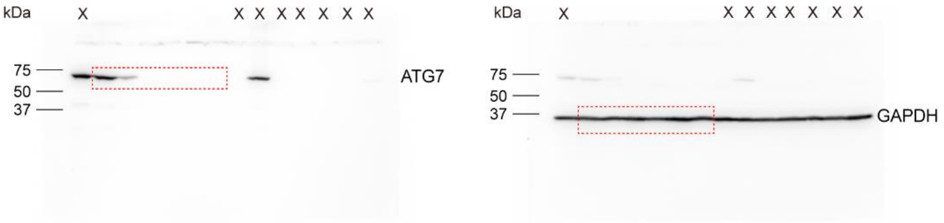

sFigure 4A

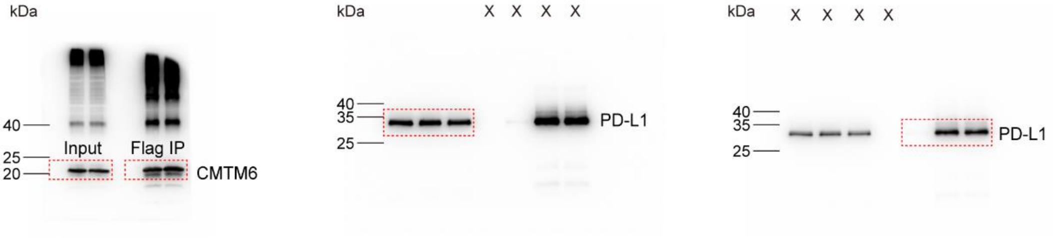

sFigure 4B

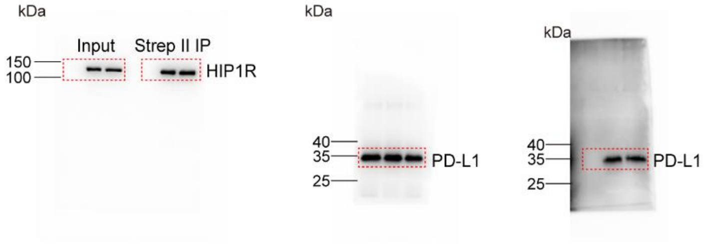

sFigure 4C

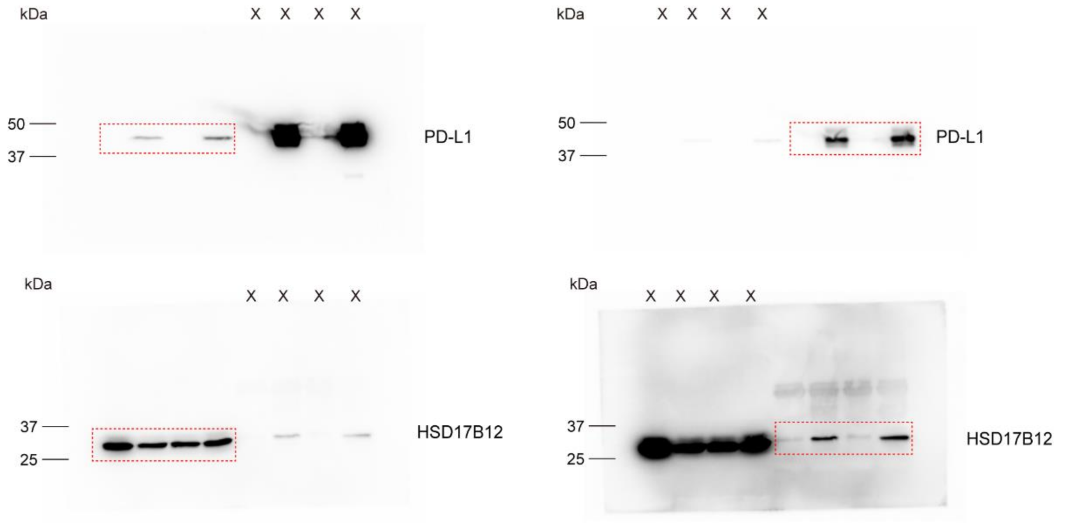

sFigure 4F

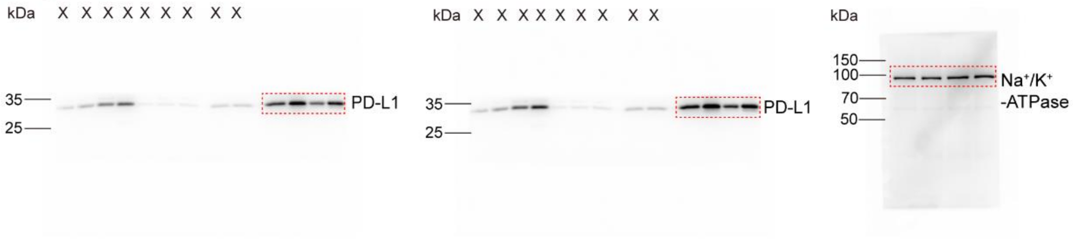

sFigure 4I

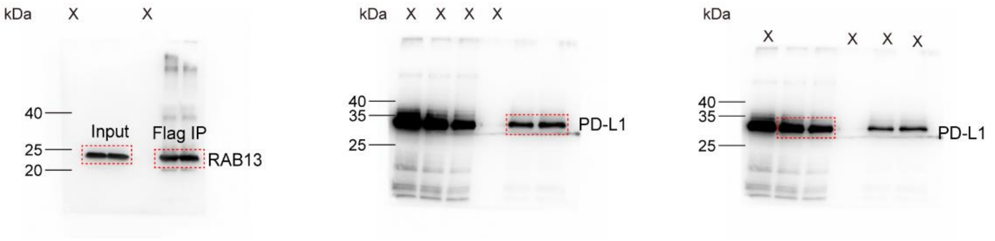

sFigure 4J

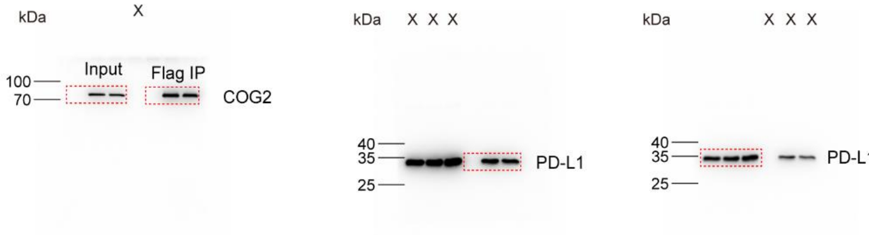

sFigure 4K

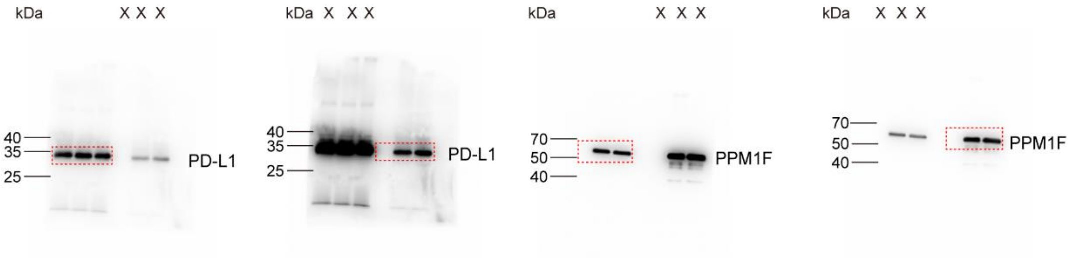

sFigure 4L

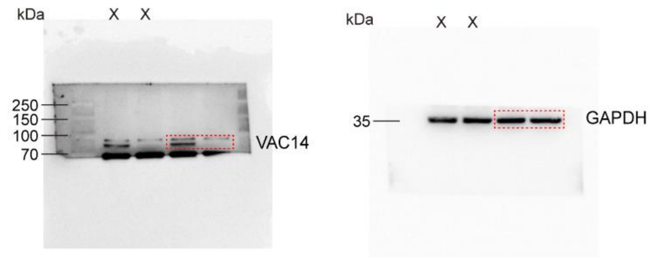

sFigure 4M

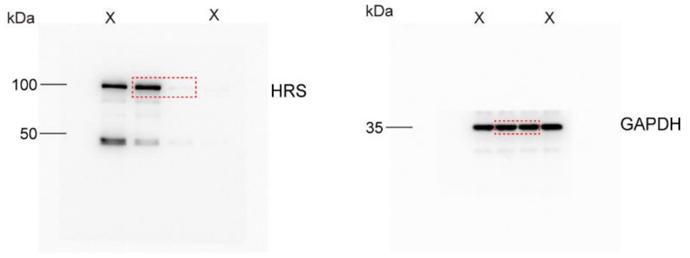

sFigure 4N

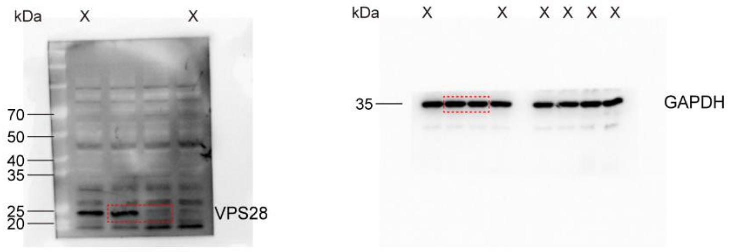

sFigure 4O

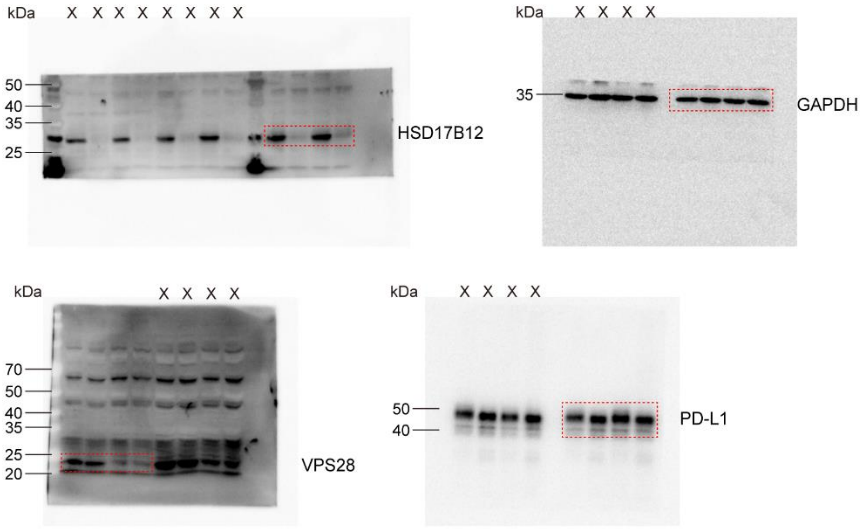

sFigure 5B

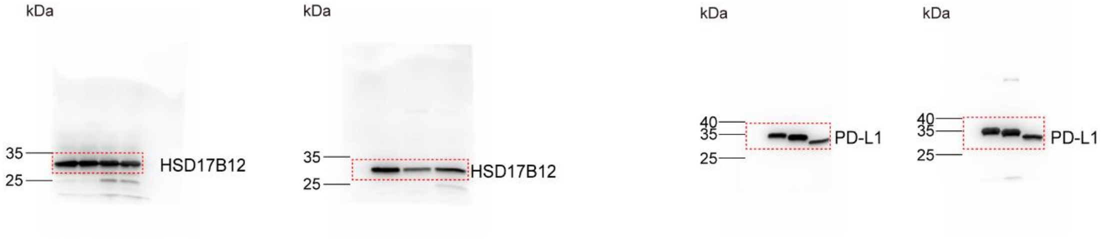

sFigure 5C

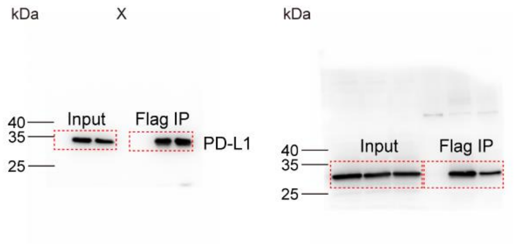

sFigure 5D

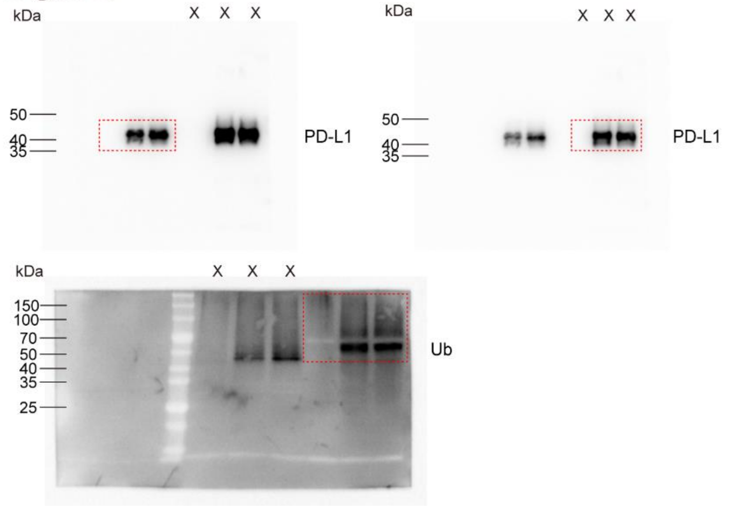

sFigure 5E

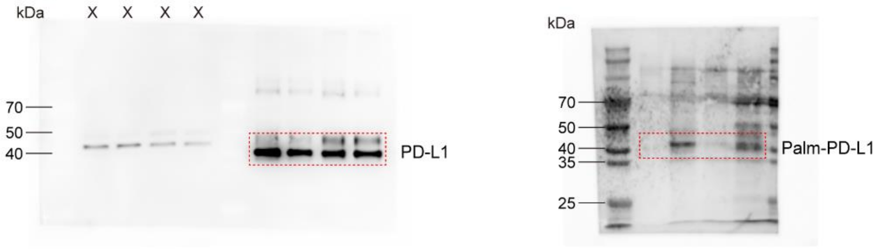

sFigure 5F

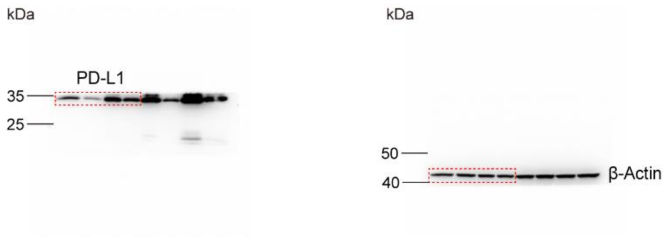

sFigure 5G

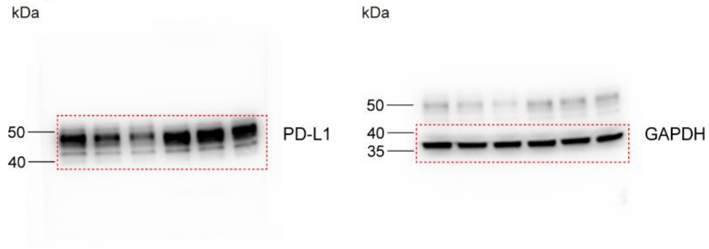

sFigure 5H

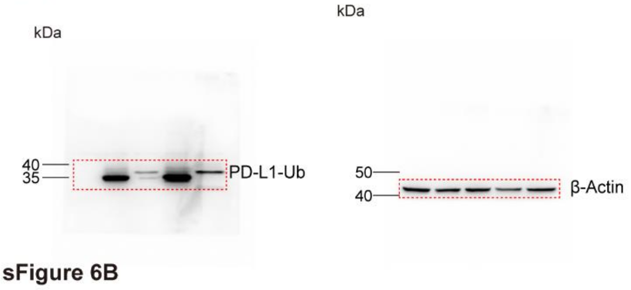

sFigure 6B

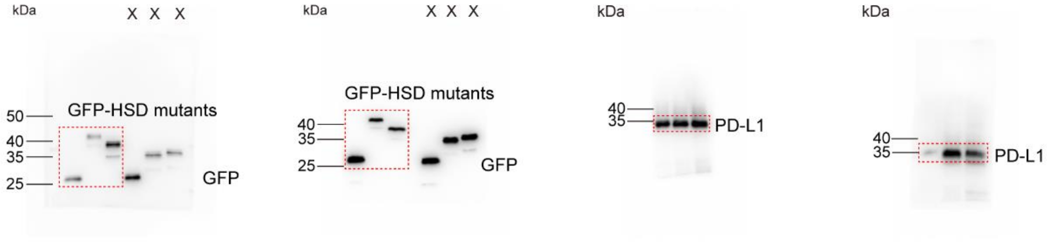

sFigure 6C

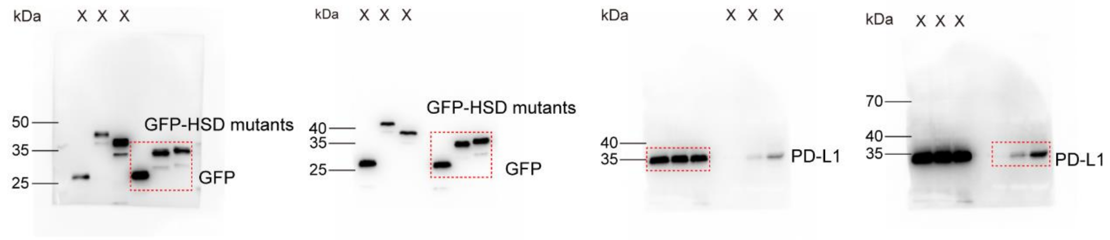

sFigure 6D

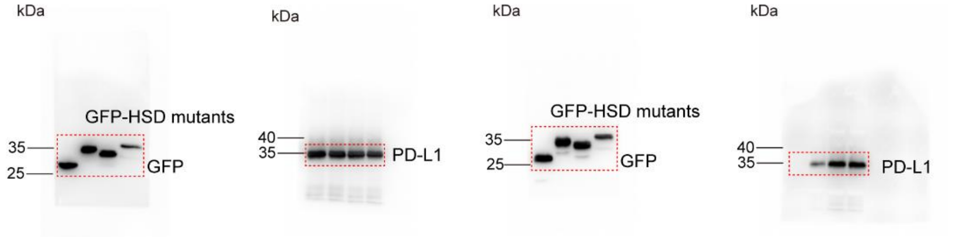

sFigure 6F

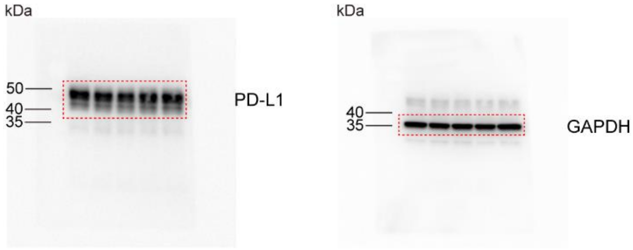

sFigure 6G

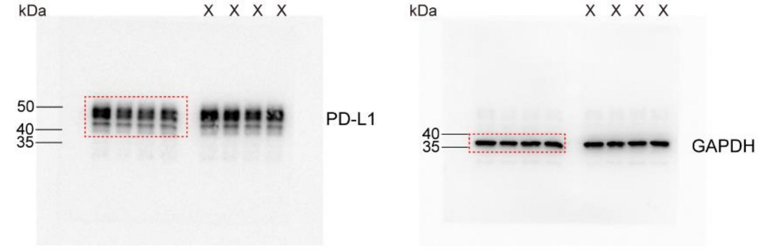

sFigure 6H

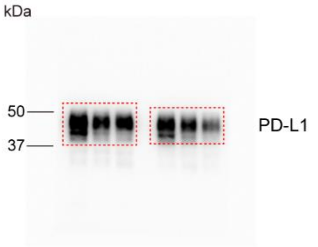

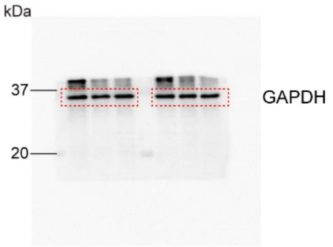

sFigure 6I

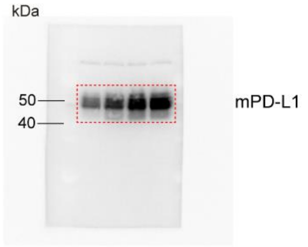

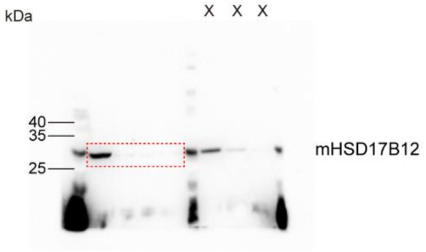

sFigure 6J

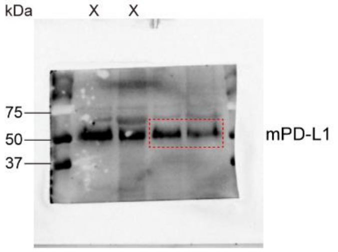

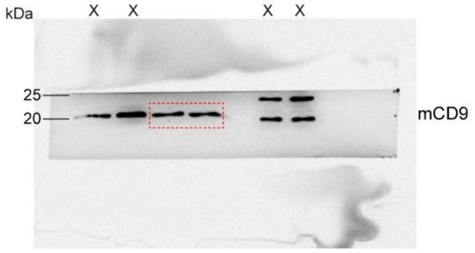

Supplement: S1 Raw Images — (PDF) [file pbio.3003603.s009.pdf]
