# Supplementary material for: The lipid-metabolic enzyme HSD17B12 drives lysosomal degradation of PD-L1 potentiating anti-tumor immunity in a mouse model
Source: PLoS Biol. 2026 Jan 27;24(1):e3003603. doi: 10.1371/journal.pbio.3003603 (PMC12843542; doi:10.1371/journal.pbio.3003603)
Supplement: S1 Table — List of HSD17B12 expression in tumor tissues and paired normal tissues. (DOCX) [file pbio.3003603.s010.docx]

**S1 Table .** Classification based on HSD17B12 relative protein expression levels

| **HSD17B12 expression in tumor tissues and paired normal tissues.** | | |  |  |
| --- | --- | --- | --- | --- |
| Sample_Normal_ID | HSD17B12 expression in normal tissues | Sample_Tumor_ID | HSD17B12 expression in tumor tissues | HSD17B12 expression classifications in tumor tissues |
| N1 | 0.371829393 | T1 | 0.350604328 | High HSD17B12 |
| N10 | 0.355233759 | T10 | 0.259773175 | High HSD17B12 |
| N100 | -0.378765391 | T100 | -0.43302441 | Low HSD17B12 |
| N101 | -0.77729182 | T101 | -0.174834344 | Low HSD17B12 |
| N102 | -0.787516436 | T102 | -0.7276226 | Low HSD17B12 |
| N103 | -0.543025391 | T103 | 0.238321758 | High HSD17B12 |
| N104 | 0.024118551 | T104 | -0.160075543 | Low HSD17B12 |
| N105 | -0.119731029 | T105 | 0.049445164 | Low HSD17B12 |
| N106 | -0.582827091 | T106 | 0.263915977 | High HSD17B12 |
| N107 | -0.362286359 | T107 | 0.151690754 | High HSD17B12 |
| N108 | -0.037601389 | T108 | 0.254281503 | High HSD17B12 |
| N109 | -0.296341443 | T109 | -0.01276934 | Low HSD17B12 |
| N11 | -0.311353924 | T11 | 0.114487283 | Low HSD17B12 |
| N111 | -1.049000598 | T111 | -0.34997137 | Low HSD17B12 |
| N12 | 0.130976473 | T12 | 0.777313883 | High HSD17B12 |
| N13 | -0.371318296 | T13 | -0.477236498 | Low HSD17B12 |
| N14 | 0.086381833 | T14 | 0.32622597 | High HSD17B12 |
| N15 | -0.056837722 | T15 | -0.094322065 | Low HSD17B12 |
| N16 | -0.362153421 | T16 | 0.25690924 | High HSD17B12 |
| N2 | 0.352756285 | T2 | 0.003370941 | Low HSD17B12 |
| N21 | -0.096626255 | T21 | 0.655573666 | High HSD17B12 |
| N22 | 0.011818491 | T22 | -0.124642219 | Low HSD17B12 |
| N23 | -0.086969769 | T23 | -0.188126157 | Low HSD17B12 |
| N24 | 0.077568392 | T24 | 0.128845273 | Low HSD17B12 |
| N25 | -0.149011794 | T25 | 0.450118956 | High HSD17B12 |
| N26 | -0.376555382 | T26 | 0.106206843 | Low HSD17B12 |
| N27 | 0.129100942 | T27 | 0.250452587 | High HSD17B12 |
| N28 | -0.125280083 | T28 | 0.217985964 | High HSD17B12 |
| N29 | -0.172714489 | T29 | -0.116711761 | Low HSD17B12 |
| N30 | 0.009591448 | T30 | -0.270745485 | Low HSD17B12 |
| N31 | -0.327654279 | T31 | 0.229237197 | High HSD17B12 |
| N32 | -0.63021084 | T32 | 0.055840926 | Low HSD17B12 |
| N33 | -0.374573505 | T33 | 0.554736204 | High HSD17B12 |
| N34 | -0.153511826 | T34 | 0.330211342 | High HSD17B12 |
| N35 | -0.13417731 | T35 | -0.405213174 | Low HSD17B12 |
| N36 | -0.00086438 | T36 | 0.388860157 | High HSD17B12 |
| N37 | 0.046768165 | T37 | 0.133779672 | High HSD17B12 |
| N38 | -0.081806743 | T38 | 0.121126719 | Low HSD17B12 |
| N39 | -0.082789666 | T39 | 0.027709346 | Low HSD17B12 |
| N4 | 0.187292121 | T4 | -0.149265569 | Low HSD17B12 |
| N40 | -0.250209958 | T40 | 0.245630674 | High HSD17B12 |
| N41 | -0.359357006 | T41 | 0.328304525 | High HSD17B12 |
| N42 | -0.196393087 | T42 | -0.027232942 | Low HSD17B12 |
| N43 | -0.744292663 | T43 | 0.385701389 | High HSD17B12 |
| N44 | -0.128279637 | T44 | -0.157081838 | Low HSD17B12 |
| N45 | -0.130691589 | T45 | 0.639702773 | High HSD17B12 |
| N46 | -0.692088099 | T46 | 0.267062254 | High HSD17B12 |
| N47 | 0.087071846 | T47 | 0.056074858 | Low HSD17B12 |
| N48 | -0.28073621 | T48 | 0.328883377 | High HSD17B12 |
| N49 | -0.625672588 | T49 | 0.031970418 | Low HSD17B12 |
| N5 | -0.630317324 | T5 | 0.405394226 | High HSD17B12 |
| N50 | -0.061245602 | T50 | 0.060421038 | Low HSD17B12 |
| N51 | -0.120186442 | T51 | 0.288841672 | High HSD17B12 |
| N52 | -0.243822837 | T52 | 0.293879872 | High HSD17B12 |
| N53 | 0.182081619 | T53 | 0.448710537 | High HSD17B12 |
| N54 | -0.651232031 | T54 | 0.812808026 | High HSD17B12 |
| N55 | 0.003148716 | T55 | -0.018876344 | Low HSD17B12 |
| N56 | 0.062811798 | T56 | 0.199145032 | High HSD17B12 |
| N57 | -0.585906367 | T57 | -0.483205057 | Low HSD17B12 |
| N58 | 0.233548104 | T58 | 0.038070607 | Low HSD17B12 |
| N59 | 0.250027248 | T59 | 0.572529338 | High HSD17B12 |
| N6 | -0.293512926 | T6 | 0.163548681 | High HSD17B12 |
| N60 | 0.31834377 | T60 | 0.352775954 | High HSD17B12 |
| N61 | -0.160046125 | T61 | 0.852522114 | High HSD17B12 |
| N62 | -0.202649195 | T62 | 0.18946712 | High HSD17B12 |
| N63 | 0.321720961 | T63 | 0.49588111 | High HSD17B12 |
| N64 | -0.412297238 | T64 | 0.348149988 | High HSD17B12 |
| N65 | -0.766816338 | T65 | -0.613585834 | Low HSD17B12 |
| N66 | -0.305367053 | T66 | -0.184876328 | Low HSD17B12 |
| N67 | -0.472884273 | T67 | 0.572387128 | High HSD17B12 |
| N68 | -0.785993685 | T68 | -0.133596857 | Low HSD17B12 |
| N69 | -0.408082964 | T69 | -0.180229353 | Low HSD17B12 |
| N7 | -0.800810634 | T7 | 0.261880841 | High HSD17B12 |
| N70 | -0.547011656 | T70 | -0.502399833 | Low HSD17B12 |
| N71 | -0.526719692 | T71 | -0.041256579 | Low HSD17B12 |
| N72 | -0.226534357 | T72 | -0.23914734 | Low HSD17B12 |
| N73 | -0.246154174 | T73 | 0.474870738 | High HSD17B12 |
| N74 | -0.121635492 | T74 | -0.045222751 | Low HSD17B12 |
| N75 | -0.018711508 | T75 | 0.099216373 | Low HSD17B12 |
| N76 | -0.350165389 | T76 | 0.159835808 | High HSD17B12 |
| N77 | -0.180679253 | T77 | 0.012691563 | Low HSD17B12 |
| N78 | -0.009828797 | T78 | -0.188109859 | Low HSD17B12 |
| N79 | -0.291632645 | T79 | 0.505606312 | High HSD17B12 |
| N8 | -0.194942326 | T8 | -0.161002472 | Low HSD17B12 |
| N80 | -0.037701768 | T80 | 0.066958777 | Low HSD17B12 |
| N81 | -0.119102489 | T81 | -0.041878556 | Low HSD17B12 |
| N82 | -0.427224599 | T82 | 0.053476366 | Low HSD17B12 |
| N83 | -0.399104385 | T83 | 0.010508486 | Low HSD17B12 |
| N84 | -0.143755477 | T84 | -0.686168887 | Low HSD17B12 |
| N85 | -0.212408512 | T85 | 0.388998313 | High HSD17B12 |
| N86 | 0.024415883 | T86 | -0.103990051 | Low HSD17B12 |
| N87 | 0.154832959 | T87 | 0.024709723 | Low HSD17B12 |
| N88 | 0.179076703 | T88 | -0.174315038 | Low HSD17B12 |
| N89 | -0.461952417 | T89 | 0.666639635 | High HSD17B12 |
| N9 | 0.126941649 | T9 | 0.105906984 | Low HSD17B12 |
| N90 | 0.198825666 | T90 | 0.291873257 | High HSD17B12 |
| N91 | -0.183524459 | T91 | 0.034532551 | Low HSD17B12 |
| N92 | -0.404051408 | T92 | 0.223383057 | High HSD17B12 |
| N93 | -0.363048 | T93 | 0.087713289 | Low HSD17B12 |
| N94 | -0.294412624 | T94 | -0.338123621 | Low HSD17B12 |
| N96 | -0.228911123 | T96 | 0.048618812 | Low HSD17B12 |
| N97 | -0.102280568 | T97 | -0.032288207 | Low HSD17B12 |
| N98 | -0.35603704 | T98 | -0.550871912 | Low HSD17B12 |
| N99 | -0.260024837 | T99 | 0.012136189 | Low HSD17B12 |
