# Supplementary material for: The lipid-metabolic enzyme HSD17B12 drives lysosomal degradation of PD-L1 potentiating anti-tumor immunity in a mouse model
Source: PLoS Biol. 2026 Jan 27;24(1):e3003603. doi: 10.1371/journal.pbio.3003603 (PMC12843542; doi:10.1371/journal.pbio.3003603)
Supplement: S2 Table — List of clinical characteristics for the colorectal cancer patients, including age, sex, overall survival, and tumor location. (DOCX) [file pbio.3003603.s011.docx]

**S2 Table.** Clinical information for CRC cohort

| Tumor  _ID | Normal  _ID | Gender | Age | OS Status (1: Dead; 0: Alive) | OS | Tumor  location | TNM | MMR  status | MSI  score | Genome | Proteome | WB | IF | IHC | |
| --- | --- | --- | --- | --- | --- | --- | --- | --- | --- | --- | --- | --- | --- | --- | --- |
| T1 | N1 | Female | 44 | 1 | 5.27 | Colon | IV | NA | 0.34 | Yes | Yes | No | No | No | |
| T2 | N2 | Female | 53 | 1 | 35 | Colon | II | NA | 0 | Yes | Yes | No | No | No | |
| T4 | N4 | Male | 42 | 1 | 23 | Colon | III | NA | 0.04 | Yes | Yes | Yes | No | No | |
| T37 | N37 | Female | 54 | 0 | 98.17 | Colon | II | NA | 9.79 | Yes | Yes | Yes | No | No | |
| T38 | N38 | Female | 76 | 0 | 98.13 | Colon | II | NA | 0.12 | Yes | Yes | Yes | No | No | |
| T39 | N39 | Male | 38 | 1 | 37 | Colon | II | NA | 0.36 | Yes | Yes | No | No | No | |
| T40 | N40 | Male | 55 | 0 | 98.03 | Colon | III | NA | 0.01 | Yes | Yes | Yes | No | No | |
| T41 | N41 | Male | 60 | 1 | 4 | Colon | III | NA | 0.03 | Yes | Yes | Yes | No | No | |
| T42 | N42 | Male | 55 | 1 | 44 | Colon | II | NA | 29.35 | Yes | Yes | No | No | No | |
| T43 | N43 | Male | 76 | 0 | 97.77 | Colon | II | NA | 0.32 | Yes | Yes | Yes | No | No | |
| T44 | N44 | Male | 57 | 0 | 97.73 | Colon | II | NA | 0.13 | Yes | Yes | Yes | No | No | |
| T45 | N45 | Male | 34 | 1 | 96.07 | Colon | II | NA | 0.17 | Yes | Yes | Yes | No | No | |
| T46 | N46 | Female | 85 | 0 | 97.27 | Colon | IV | NA | 0.04 | Yes | Yes | Yes | No | No | |
| T47 | N47 | Male | 73 | 1 | 28 | Colon | III | NA | 0.2 | Yes | Yes | Yes | No | No | |
| T48 | N48 | Female | 60 | 0 | 97.2 | Colon | III | NA | 0.09 | Yes | Yes | Yes | No | No | |
| T49 | N49 | Male | 66 | 0 | 96.83 | Colon | II | NA | 0.2 | Yes | Yes | No | No | No | |
| T50 | N50 | Female | 56 | 1 | 33 | Colon | III | NA | 0.08 | Yes | Yes | Yes | No | No | |
| T51 | N51 | Female | 41 | 0 | 96.6 | Colon | III | NA | 0.55 | Yes | Yes | Yes | No | No | |
| T52 | N52 | Female | 48 | 0 | 96.3 | Colon | II | NA | 0.19 | Yes | Yes | Yes | No | No | |
| T53 | N53 | Male | 65 | 0 | 96.1 | Colon | I | NA | 0.31 | Yes | Yes | Yes | No | No | |
| T54 | N54 | Female | 71 | 0 | 95.8 | Colon | I | NA | 0.38 | Yes | Yes | Yes | No | No | |
| T55 | N55 | Male | 77 | 1 | 53 | Colon | III | NA | 0.23 | Yes | Yes | No | No | No | |
| T56 | N56 | Male | 51 | 0 | 95.6 | Colon | II | NA | 22.35 | Yes | Yes | No | No | No | |
| T57 | N57 | Female | 39 | 0 | 95.47 | Colon | II | NA | 0.07 | Yes | Yes | Yes | No | No | |
| T58 | N58 | Male | 74 | 1 | 14 | Colon | IV | NA | 0.1 | Yes | Yes | Yes | No | No | |
| T59 | N59 | Female | 78 | 1 | 11 | Colon | III | NA | 0.43 | Yes | Yes | Yes | No | No | |
| T60 | N60 | Female | 54 | 1 | 62 | Colon | II | NA | 0.78 | Yes | Yes | No | No | No | |
| T61 | N61 | Female | 77 | 1 | 51.3 | Colon | III | NA | 0.35 | Yes | Yes | Yes | No | No |  |
| T62 | N62 | Female | 56 | 1 | 61 | Colon | III | NA | 0.19 | Yes | Yes | Yes | No | No |  |
| T63 | N63 | Male | 71 | 0 | 89.83 | Colon | II | NA | 11.36 | Yes | Yes | Yes | No | No |  |
| T64 | N64 | Female | 76 | 0 | 89.37 | Colon | III | NA | 0.3 | Yes | Yes | Yes | No | No |  |
| T65 | N65 | Female | 68 | 0 | 89.37 | Colon | IV | NA | 1.46 | Yes | Yes | Yes | No | No |  |
| T66 | N66 | Female | 56 | 0 | 89.13 | Colon | III | NA | 0.34 | Yes | Yes | No | No | No |  |
| T67 | N67 | Female | 74 | 1 | 1.93 | Colon | III | NA | 0.03 | Yes | Yes | No | No | No |  |
| T68 | N68 | Female | 45 | 1 | 25 | Colon | II | NA | NA | No | Yes | No | No | No |  |
| T69 | N69 | Female | 60 | 1 | 32.37 | Colon | III | NA | 0.21 | Yes | Yes | Yes | No | No |  |
| T70 | N70 | Male | 56 | 1 | 24 | Colon | III | NA | 0.05 | Yes | Yes | Yes | No | No |  |
| T71 | N71 | Male | 54 | 0 | 84.7 | Colon | III | NA | 0.39 | Yes | Yes | Yes | No | No |  |
| T72 | N72 | Female | 66 | 1 | 11.47 | Colon | IV | NA | 0.13 | Yes | Yes | Yes | No | No |  |
| T73 | N73 | Male | 74 | 1 | 13 | Colon | III | NA | 0.42 | Yes | Yes | Yes | No | No |  |
| T74 | N74 | Female | 66 | 1 | 12 | Colon | III | NA | 0 | Yes | Yes | Yes | No | No |  |
| T75 | N75 | Male | 54 | 1 | 26 | Colon | III | NA | 20.97 | Yes | Yes | No | No | No |  |
| T76 | N76 | Female | 36 | 0 | 64.57 | Rectum | NA | NA | NA | No | Yes | No | No | No |  |
| T5 | N5 | Female | 69 | 0 | 107.57 | Colon | III | NA | 0.1 | Yes | Yes | Yes | No | No |  |
| T6 | N6 | Female | 52 | 0 | 106.9 | Colon | II | NA | 0.03 | Yes | Yes | Yes | No | No |  |
| T7 | N7 | Male | 76 | 0 | 106.8 | Colon | III | NA | 0.33 | Yes | Yes | Yes | No | No |  |
| T8 | N8 | Male | 63 | 1 | 16.6 | Colon | III | NA | 0.06 | Yes | Yes | No | No | No |  |
| T77 | N77 | Female | 36 | 0 | 64.67 | Rectum | III | NA | NA | No | Yes | Yes | No | No |  |
| T78 | N78 | Male | 38 | 0 | 65.73 | Rectum | III | NA | NA | No | Yes | Yes | No | No |  |
| T79 | N79 | Male | 26 | 0 | 66.4 | Rectum | III | NA | NA | No | Yes | Yes | No | No |  |
| T80 | N80 | Female | 37 | 0 | 66.73 | Rectum | III | NA | NA | No | Yes | No | No | No |  |
| T81 | N81 | Female | 38 | 0 | 66.87 | Rectum | III | NA | NA | No | Yes | No | No | No |  |
| T82 | N82 | Male | 30 | 0 | 67.63 | Rectum | II | NA | NA | No | Yes | No | No | No |  |
| T83 | N83 | Male | 36 | 1 | 43.1 | Rectum | II | NA | NA | No | Yes | Yes | No | No |  |
| T84 | N84 | Male | 23 | 0 | 67.93 | Rectum | II | NA | NA | No | Yes | Yes | No | No |  |
| T85 | N85 | Female | 28 | 0 | 68.13 | Rectum | III | NA | NA | No | Yes | Yes | No | No |  |
| T86 | N86 | Male | 30 | 1 | 11.77 | Rectum | III | NA | NA | No | Yes | No | No | No |  |
| T87 | N87 | Male | 40 | 1 | 22.27 | Rectum | III | NA | NA | No | Yes | No | No | No |  |
| T88 | N88 | Male | 33 | 0 | 68.6 | Rectum | III | NA | NA | No | Yes | No | No | No |  |
| T89 | N89 | Female | 40 | 0 | 72.57 | Rectum | III | NA | NA | No | Yes | Yes | No | No |  |
| T90 | N90 | Male | 38 | 0 | 72.8 | Rectum | III | NA | NA | No | Yes | No | No | No |  |
| T91 | N91 | Female | 39 | 0 | 73.2 | Rectum | II | NA | NA | No | Yes | Yes | No | No |  |
| T92 | N92 | Female | 36 | 1 | 6.67 | Rectum | III | NA | NA | No | Yes | No | No | No |  |
| T93 | N93 | Male | 39 | 1 | 34.2 | Rectum | II | NA | NA | No | Yes | Yes | No | No |  |
| T94 | N94 | Male | 22 | 0 | 77.67 | Rectum | III | NA | NA | No | Yes | No | No | No |  |
| T96 | N96 | Male | 34 | 0 | 79.6 | Rectum | III | NA | NA | No | Yes | Yes | No | No |  |
| T100 | N100 | Female | 35 | 0 | 83.27 | Rectum | II | NA | NA | No | Yes | No | No | No |  |
| T97 | N97 | Male | 40 | 1 | 18.77 | Rectum | III | NA | NA | No | Yes | Yes | No | No |  |
| T98 | N98 | Male | 26 | 1 | 17.07 | Rectum | III | NA | NA | No | Yes | No | No | No |  |
| T99 | N99 | Male | 36 | 1 | 36.73 | Colon | III | NA | NA | No | Yes | No | No | No |  |
| T101 | N101 | Male | 40 | 0 | 83.47 | Rectum | I | NA | NA | No | Yes | No | No | No |  |
| T102 | N102 | Male | 38 | 1 | 80.5 | Rectum | III | NA | NA | No | Yes | No | No | No |  |
| T103 | N103 | Female | 28 | 0 | 87.9 | Rectum | I | NA | NA | No | Yes | No | No | No |  |
| T104 | N104 | Male | 40 | 1 | 21.23 | Rectum | III | NA | NA | No | Yes | No | No | No |  |
| T105 | N105 | Male | 39 | 0 | 64.3 | Colon | NA | NA | NA | No | Yes | No | No | No |  |
| T106 | N106 | Female | 33 | 1 | 31 | Colon | II | NA | NA | No | Yes | No | No | No |  |
| T107 | N107 | Female | 30 | 0 | 69.93 | Colon | III | NA | NA | No | Yes | No | No | No |  |
| T108 | N108 | Male | 35 | 0 | 73.2 | Colon | II | NA | NA | No | Yes | No | No | No |  |
| T109 | N109 | Female | 39 | 1 | 21 | Colon | I | NA | NA | No | Yes | No | No | No |  |
| T111 | N111 | Male | 33 | 0 | 75.5 | Colon | I | NA | NA | No | Yes | No | No | No |  |
| T10 | N10 | Male | 82 | 1 | 5.57 | Colon | I | NA | 0.14 | Yes | Yes | Yes | No | No |  |
| T11 | N11 | Female | 82 | 1 | 15.43 | Colon | II | NA | 3.56 | Yes | Yes | Yes | No | No |  |
| T12 | N12 | Female | 62 | 0 | 105.23 | Colon | III | NA | 0.31 | Yes | Yes | Yes | No | No |  |
| T9 | N9 | Male | 42 | 1 | 15 | Colon | II | NA | 10.11 | Yes | Yes | No | No | No |  |
| T13 | N13 | Male | 65 | 1 | 13 | Colon | IV | NA | 0.01 | Yes | Yes | Yes | No | No |  |
| T14 | N14 | Male | 71 | 0 | 103.3 | Colon | III | NA | 0.05 | Yes | Yes | Yes | No | No |  |
| T15 | N15 | Female | 91 | 0 | 102.33 | Colon | III | NA | 31.19 | Yes | Yes | No | No | No |  |
| T16 | N16 | Female | 40 | 0 | 101.97 | Colon | I | NA | 28.55 | Yes | Yes | Yes | No | No |  |
| T21 | N21 | Female | 60 | 1 | 7 | Colon | III | NA | 0.4 | Yes | Yes | Yes | No | No |  |
| T22 | N22 | Male | 33 | 1 | 6.1 | Colon | III | NA | 0.12 | Yes | Yes | Yes | No | No |  |
| T23 | N23 | Male | 46 | 1 | 32 | Colon | II | NA | 0.85 | Yes | Yes | Yes | No | No |  |
| T24 | N24 | Male | 43 | 1 | 11.8 | Colon | IV | NA | 35.65 | Yes | Yes | No | No | No |  |
| T25 | N25 | Male | 62 | 0 | 99.13 | Colon | I | NA | 0.51 | Yes | Yes | Yes | No | No |  |
| T26 | N26 | Male | 53 | 0 | 99.07 | Colon | II | NA | 0.31 | Yes | Yes | Yes | No | No |  |
| T27 | N27 | Male | 67 | 1 | 23 | Colon | III | NA | 0.84 | Yes | Yes | No | No | No |  |
| T28 | N28 | Male | 39 | 1 | 37 | Colon | III | NA | 0.05 | Yes | Yes | Yes | No | No |  |
| T29 | N29 | Male | 51 | 0 | 98.87 | Colon | III | NA | 0.22 | Yes | Yes | Yes | No | No |  |
| T30 | N30 | Female | 56 | 1 | 1.5 | Colon | IV | NA | 0.6 | Yes | Yes | No | No | No |  |
| T31 | N31 | Male | 45 | 0 | 98.7 | Colon | II | NA | 0.14 | Yes | Yes | Yes | No | No |  |
| T32 | N32 | Female | 78 | 1 | 27 | Colon | III | NA | 0.01 | Yes | Yes | Yes | No | No |  |
| T33 | N33 | Male | 68 | 0 | 98.47 | Colon | I | NA | 0.18 | Yes | Yes | Yes | No | No |  |
| T34 | N34 | Male | 50 | 1 | 72.7 | Colon | II | NA | 27.98 | Yes | Yes | Yes | No | No |  |
| T35 | N35 | Male | 60 | 1 | 61.23 | Colon | III | NA | 0.17 | Yes | Yes | Yes | No | No |  |
| T36 | N36 | Male | 64 | 1 | 62 | Colon | II | NA | 1.15 | Yes | Yes | Yes | No | No |  |
| T112 |  | Male | 50 | 0 | 63.33 | Colon | I | pMMR | NA | No | No | Yes | Yes | Yes |  |
| T113 |  | Male | 62 | 1 | 42.47 | Colon | II | pMMR | NA | No | No | Yes | No | No |  |
| T114 |  | Female | 86 | 0 | 61.87 | Colon | II | dMMR | NA | No | No | Yes | Yes | Yes |  |
| T115 |  | Female | 44 | 0 | 64.5 | Colon | II | dMMR | NA | No | No | Yes | No | No |  |
| T116 |  | Male | 46 | 0 | 64.43 | Colon | II | dMMR | NA | No | No | Yes | Yes | Yes |  |
| T117 |  | Female | 61 | 1 | 15.5 | Colon | III | pMMR | NA | No | No | Yes | No | No |  |
| T118 |  | Female | 59 | 0 | 64.2 | Colon | III | pMMR | NA | No | No | Yes | Yes | Yes |  |
| T119 |  | Female | 66 | 0 | 63.8 | Colon | II | pMMR | NA | No | No | Yes | No | No |  |
| T120 |  | Female | 52 | 0 | 63.57 | Colon | II | dMMR | NA | No | No | Yes | Yes | Yes |  |
| T121 |  | Male | 30 | 1 | 25.97 | Colon | III | pMMR | NA | No | No | Yes | No | No |  |
| T122 |  | Female | 48 | 0 | 65.17 | Colon | III | NA | NA | No | No | Yes | Yes | No |  |
| T123 |  | Male | 37 | 0 | 64.97 | Colon | III | pMMR | NA | No | No | Yes | Yes | Yes |  |
| T124 |  | Male | 32 | 1 | 64.97 | Colon | II | dMMR | NA | No | No | Yes | Yes | Yes |  |
| T125 |  | Male | 53 | 0 | 65.2 | Colon | I | pMMR | NA | No | No | Yes | No | No |  |
| T126 |  | Male | 70 | 1 | 48.17 | Colon | III | pMMR | NA | No | No | Yes | No | No |  |
| DM1 |  | Female | 35 | 0 | 16.43 | Colon | I | dMMR | NA | No | No | No | No | Yes |  |
| DM2 |  | Female | 30 | 0 | 29.57 | Colon | III | dMMR | NA | No | No | No | No | Yes |  |
| DM3 |  | Female | 50 | 0 | 25.4 | Colon | II | dMMR | NA | No | No | No | No | Yes |  |
| DM4 |  | Male | 27 | 0 | 11.53 | Colon | III | dMMR | NA | No | No | No | No | Yes |  |
| DM5 |  | Male | 63 | 0 | 7.93 | Colon | II | dMMR | NA | No | No | No | No | Yes |  |
| DM6 |  | Male | 30 | 0 | 1.8 | Colon | I | dMMR | NA | No | No | No | No | Yes |  |
| DM7 |  | Male | 58 | 0 | 2.83 | Colon | II | dMMR | NA | No | No | No | No | Yes |  |

Abbreviations: OS--Overall survival; TNM--Tumor node metastasis; MMR--Mismatch Repair; MSI--Microsatellite Instability; WB-Western Blotting; IF--Immunofluorescence; IHC--Immunohistochemistry.
